# Adiponectin receptor T-cadherin emerges as a novel regulator of adipose stem cell quiescence and adipogenesis

**DOI:** 10.3389/fcell.2025.1734183

**Published:** 2026-01-15

**Authors:** V. Yu Sysoeva, M. S. Arbatsky, K. Yu Kulebyakin, P. A. Tyurin-Kuzmin, E. V. Semina, P. S. Klimovich, I. B. Brodsky, D. D. Romashin, A. F. Altyeva, N. S. Voloshin, N. I. Kalinina, M. A. Vigovskiy, V. A. Tkachuk, K. A. Rubina

**Affiliations:** Faculty of Medicine, Lomonosov Moscow State University, Moscow, Russia

**Keywords:** adipogenesis, adiponectin receptor, AD-MSCs, DPP4, LDL, single-cell RNA sequencing, T-cadherin

## Abstract

The question of heterogeneity among adipose tissue cells and mesenchymal stem/stromal cells mesenchymal stem cells derived from white adipose tissue has long been a subject of interest. In our study, we conducted a comprehensive single-cell RNA-seq analysis on MSCs isolated from human subcutaneous adipose tissue and maintained under control conditions or upon adipogenic induction. Our findings unveiled a distinct subpopulation of T-cadherin expressing cells, which co-expressed Dipeptidyl peptidase–4 (DPP4^+^), a marker of multipotent progenitors in the adipose tissue. Moreover, T-cadherin co-expressed with DPP4^+^ in early progenitors both, *in vivo* and *in vitro*. While adipogenic induction resulted in overall T-cadherin decline, in both the control and differentiated samples, there existed cells with high T-cadherin concurrently expressing stemness-related genes. Pseudotemporal trajectories analysis based on the scRNA-seq data, revealed that T-cadherin-expressing cells constituted a discrete cell subpopulation with stem-like properties, rather than participating in adipogenic differentiation. Using lentiviral transduction, we manipulated T-cadherin expression in MSCs and found that cells overexpressing T-cadherin also displayed an elevated level of DPP4. Strikingly, these cells exhibited significantly slower rates of proliferation compared to the controls. Long-term live-cell imaging, which allowed for the tracking of individual cell fates over a span of 10 days, together with classical adipogenic differentiation assays, revealed a substantial reduction in adipogenic differentiation capacity among MSCs overexpressing T-cadherin. Consequently, our experimental findings support a compelling model wherein T-cadherin, positioned upstream of DPP4, plays a pivotal role in maintaining a stem-like status within a distinct subpopulation of T-cadherin-expressing cells within MSCs.

## 1 Introduction

Adipose tissue is a key regulator of systemic energy balance and endocrine function. In white adipose tissue (WAT), excess energy is stored as triglycerides within mature adipocytes and mobilized during energy deprivation as free fatty acids to meet systemic energy demands. Under sustained overnutrition, WAT expands to increase storage capacity and prevent ectopic lipid deposition and lipotoxicity in peripheral organs. Beyond energy storage, WAT also functions as an active endocrine organ, secreting a broad array of biologically active molecules—including hormones, adipokines, extracellular matrix proteins, growth factors, and microRNAs—that regulate appetite, metabolism, and tissue homeostasis (Kahn et al., 2019; Vishvanath and Gupta, 2019). Key adipokines and hormones such as leptin, adiponectin, resistin, retinol-binding protein 4 (RBP4), apelin, omentin, nesfatin-1, and asprosin play essential roles in controlling satiety, energy utilization, and insulin sensitivity (Kahn et al., 2019; Kusminski et al., 2016). WAT expands and remodels in a manner that has direct consequences for metabolic health. Visceral WAT accumulation is strongly associated with insulin resistance, whereas subcutaneous WAT expansion is generally protective.

Pathological remodeling of WAT, characterized by adipocyte hypertrophy, chronic inflammation, and fibrosis, is a key driver of adipose tissue dysfunction and insulin resistance. By contrast, “healthy” WAT expansion observed in metabolically healthy obese individuals, is associated with smaller, more numerous adipocytes and reduced inflammation.

A hallmark of WAT is its remarkable plasticity, which is manifested through the presence of resident stem/progenitor cells and committed preadipocytes that undergo controlled adipogenic differentiation. Stem/progenitor cells play a pivotal role in the continuous generation and renewal of adipocytes. Physiological adipose tissue expansion is characterized by increased vascularity and adipocyte formation through differentiation of the residential stem/progenitor cells, a process known as hyperplasia (Cristancho and Lazar, 2011; Crewe et al., 2017). By contrast, obesity often promotes adipocyte hypertrophy, accompanied by chronic inflammation and fibrosis in white adipose tissue (WAT), which contributes to the development of insulin resistance, type 2 diabetes, and cardiovascular disease (Vishvanath and Gupta, 2019; Kusminski et al., 2016; Cristancho and Lazar, 2011; Neeland et al., 2019). Therefore, understanding the cellular composition and mechanisms underlying the formation of new adipocytes is crucial, yet remains incompletely understood.

A defining characteristic of pathological WAT remodeling is perturbed adipokine secretion, which ultimately affects the systemic metabolism. One of the most important adipokines secreted by white adipocytes is adiponectin (Scherer et al., 1995; Whitehead et al., 2006) with the most metabolically active form being high-molecular-weight (HMW) adiponectin (Rubina et al., 2021). Adiponectin exerts numerous protective effects in various tissues and organs including insulin-sensitizing, anti-inflammation, anti-proliferation, and anti-atherosclerotic actions (Achari and Jain, 2017). Adiponectin levels decline in pathological conditions such as insulin resistance, type 2 diabetes mellitus (T2DM), obesity, metabolic syndrome, and cardiovascular diseases with numerous studies demonstrating an inverse correlation between adiponectin levels and obesity, where weight loss is accompanied by increased circulating HMW adiponectin content (Rubina et al., 2021). Adiponectin can interact with the canonical AdipoR1 and AdipoR2 receptors, as documented in previous studies (Achari and Jain, 2017; Yamauchi et al., 2003). However, T-cadherin has been recognized to be an exclusive receptor for HMW adiponectin, yet the significance of this binding and downstream signaling pathways remains largely unexplored (Hug et al., 2004; Fukuda et al., 2021).

T-cadherin, also known as cadherin-13 (H-cadherin), is encoded by the *CDH13* gene. T-cadherin and adiponectin co-localize in various organs and tissues, with T-cadherin playing a pivotal role in mediating the protective effects of adiponectin. Using an ischemia-reperfusion injury model, Denzel et al., demonstrated that mice lacking T-cadherin had an increased infarct size similar to that observed in adiponectin knockout mice. Adenovirus-mediated adiponectin expression rescued the phenotype of adiponectin-deficient mice, yet had no effect in T-cadherin deficient mice (Denzel et al., 2010). Similar findings were obtained in the context of skeletal muscles using the hind limb revascularization murine model (Parker-Duffen et al., 2013). Both, T-cadherin-deficient and adiponectin-deficient mice exhibited impaired blood flow recovery compared to the control group. However, the introduction of exogenous adiponectin restored the phenotype only in adiponectin-deficient mice (Parker-Duffen et al., 2013).

While T-cadherin expression in cardiovascular and nervous systems has been well-documented (Rubina et al., 2021), its function within adipose tissue largely remains largely unknown. In individuals with obesity and in obese mice, both the tissue content and plasma levels of T-cadherin are markedly diminished and can be restored upon weight reduction (Göddeke et al., 2018).

Recent advances applying single-cell transcriptomics enabled detailed studies of tissue cell composition and biological processes, shedding light on cellular differentiation at the individual cell level. Single-cell RNA-seq analysis unequivocally affirmed the presence of T-cadherin in mesenchymal stem/stromal cells (MSCs) in adipose tissue. Specifically, T-cadherin mRNA was identified in MSC cell clusters concurrently expressing mRNAs for PDGFRα (Pdgfrα) and Meflin (Islr) (Nakamura et al., 2020), the latter being specific markers of MSC-like cells or mesenchymal progenitors (Challa et al., 2015; Merrick et al., 2019; Schwalie et al., 2018; Burl et al., 2018). While the data mentioned above, along with the other published findings (as reviewed in Rubina et al. (2021)), suggest a conceivable role for T-cadherin in MSCs of varying origins (Nakamura et al., 2020; Challa et al., 2015; Holley et al., 2015; Ramirez et al., 2020), robust experimental evidence elucidating the role of T-cadherin in WAT is lacking.

In the experiments detailed below, we performed comprehensive and unbiased scRNA-seq of mesenchymal stem/stromal cells from human subcutaneous adipose tissue maintained in the control conditions or after a 4-day induction of adipogenic differentiation. Our results revealed the presence of a distinct subpopulation of T-cadherin-expressing MSCs. Notably, these cells co-expressed Dipeptidyl peptidase–4 (DPP4^+^), a well-known marker associated with multipotent progenitors in white adipose tissue (WAT), along with other genes related to stemness (Merrick et al., 2019). The method applied to construct the pseudotemporal trajectory from scRNA-seq data confirmed these results suggesting that T-cadherin-expressing cells can contribute to the pool of undifferentiated cycling cells with stem-like properties. In human WAT, T-cadherin-expressing cells were found in close proximity to blood vessels or within the interstitium. To further elucidate the role of T-cadherin, we conducted experiments involving lentiviral construct to alter T-cadherin expression. We demonstrated that T-cadherin acts upstream of DPP4. Moreover, T-cadherin overexpressing MSCs exhibited distinctive properties including a reduced ability to differentiate into adipocytes and a diminished proliferative potential. Overall, our data offer new insights into cellular complexity and regulatory mechanisms within adipose tissue, shedding light on the role of T-cadherin in cell renewal and differentiation.

In the present study, we performed a comprehensive analysis to elucidate the role of T-cadherin in human adipose tissue biology. We integrated single-cell RNA sequencing (scRNA-seq) and prior biological knowledge of MSC subset-specific markers with immunofluorescent staining, flow cytometry, quantitative RT-PCR, Western blotting, lentiviral overexpression, real-time live-cell imaging, neural network–based quantification of differentiating adipocytes, and pseudotemporal trajectory analysis. Through this multifaceted approach, we systematically characterized the molecular and functional properties of T-cadherin expressing MSC subpopulations. These complementary methods enabled cross-validation of our findings across independent platforms and provided mechanistic insights into how T-cadherin may regulate cell renewal, proliferation, and adipogenic differentiation in adipose tissue.

## 2 Materials and methods

### 2.1 Cell culture

Human adipose tissue-derived mesenchymal stem cells (MSCs) from healthy donors were obtained through the live system repository collection (https://human.depo.msu.ru/#). All donors provided informed consent, and the study was approved by the local ethics committee of the Medical Research Education Center of Lomonosov Moscow State University (IRB00010587, Moscow, Russia), protocol (#160, 22 July 2019, and #4, 4 June 2018, respectively). In total, 15 donor-derived MSC lines were enrolled in this study.

Cells were isolated, cultured, and charaNcterized as previously described (Kalinina et al., 2015) in accordance with the minimal criteria for defining MSCs established by the International Society for Cellular Therapy (ISCT) (Dominici et al., 2006). Primary MSCs were cultured in AdvanceSTEM Mesenchymal Stem Cell Media containing 10% AdvanceSTEM Supplement (HyClone, Cytiva, United States) and 1% antibiotic–antimycotic solution (HyClone, Cytiva, United States) at 37 °C in a 5% CO_2_ incubator (Binder, Tuttlingen, Germany, CB210). Cells were passaged at 70%–80% confluency using Versen solution (Paneco, Russia) and HyQTase (HyClone, Cytiva, United States). For the experiments, MSCs within four to five passages were used.

### 2.2 Viral transduction of MSCs

For T-cadherin overexpression, two types of lentiviral constructs were used depending on the specific experimental design: LV-LeGO-hTcad-IRES-eGFP with its corresponding control LV-LeGO-eGFP previously generated in our laboratory (Sysoeva et al., 2024), or pLJM-Tcad–mKate with the control pRPV-contr (Eurogene, Russia).

To this end, MSCs of the two to three passages were seeded at a concentration of 100,000 cells/mL on 12-well plates. To this end, a subconfluent monolayer of MSCs was treated with 1 mL suspension containing lentiviral particles at a concentration of 3.0 × 10^5^ TU/mL for T-cadherin overexpression and 2.0 × 10^5^ TU/mL for control virus, with protamine sulfate added at 50 μg/mL to enhance transduction efficiency. Subsequently, 0.5 mL of AdvanceSTEM Mesenchymal Stem Cell Medium supplemented with 10% AdvanceSTEM Supplement and 1% antibiotic–antimycotic solution (both HyClone, Cytiva, United States) was added to each well. After 24 h, mKate or GFP fluorescence in individual cells was visualized and recorded. Images were acquired with a microscope Leica DMI 6000B equipped with Leica DFC7000T digital camera using LAS X software (Leica).

To obtain a homogeneous population, the cells were sorted using a FACS Aria III cell sorter (BD, Franklin Lakes, NJ, United States) and subsequently expanded in AdvanceSTEM Mesenchymal Stem Cell Medium supplemented with 10% AdvanceSTEM Supplement and 1% antibiotic–antimycotic solution (both HyClone, Cytiva, United States). A total of eight donor-derived cell lines were used in this study.

### 2.3 Cell processing for scRNA-seq

MSCs of the two to three passages were detached from culture dishes using HyQTase Detachment Reagent (HyClone, GE Healthcare Life Sciences, United States) and loaded onto each lane at a concentration of 10,000 cells. scRNA-seq libraries were generated using Chromium Next GEM Single Cell 3′ GEM, Library and Gel Bead Kit (10x Genomics) chemistry. The libraries were sequenced on a HiSeq1500 (Illumina) and counted using Cell Ranger v7.0 (10x Genomics) with the GRCh38 human genome. The read configuration for the libraries was 2 × 150 bp paired-end. The average number of reads per sample was 300 million, with a sequencing depth of 1.2 billion read pairs.

### 2.4 Bioinformatic data analysis

The resulting reads were aligned, and gene-level unique molecular identifier (UMI) counts were obtained by using Cell Ranger (Pipeline). Plots were generated using the R package Seurat (v. 4.1.0) by displaying cells from the control sample or experimental cell sample after induction for adipogenic differentiation within 4 days. Split violin-plots were created using the R package Seurat applying VlnPlot function with split.by = “sample” argument. T-cadherin expression level (*CDH13 gene*) was visualized using the FeaturePlot function. The percentage of cells expressing T-cadherin was evaluated utilizing the Percent_Expressing function. The integrated SeuratObject of control cells and cells induced for adipogenic differentiation was used as the input for trajectory analysis. The dynwrap, dyno, tidyverse, dyndimred, and dynplot R packages from the dynverse collection were applied for trajectory analysis. To construct trajectories, the paga_tree method was selected from the tree methods. Since the method requires the specification of prior information, namely, start_id and end_id, a random cell from a presumptive cluster was chosen as a start_id as a starting point for trajectory construction. As an end_id, a random cell from a presumptive cluster was selected as an end point for trajectory construction. Overall, this approach allows for constructing a trajectory passing through all the clusters from a starting point to an end point. For the control sample, the following subset of parameters was applied: subset (obj, subset = nFeature_RNA > 2,000 and nFeature_RNA < 6,000 and nCount_RNA > 7,000 and nCount_RNA < 30,000 and percent.mt < 10). For the cells in the adipogenic sample, the following subset of parameters was applied: subset (obj, subset = nFeature_RNA > 2,500 and nFeature_RNA < 7,500 and nCount_RNA > 7,000 and nCount_RNA < 50,000 and percent.mt < 10).

The CCA (canonical correlation analysis) method was enrolled for data integration. To obtain the heatmap of stemness genes, the output.cloupe file from the cellranger count pipeline CellRanger was used. Subsequently, cellranger aggr was used to obtain the.aggr file. Two separate datasets were created–cells with high T-cadherin expression (more than 1-fold change of the average expression level in the sample) and cells with low T-cadherin (less than 1-fold change). For each dataset, heatmaps were generated for stemness genes.

Cell type annotation was performed both, by manually assigning a cell type to each cluster and using the automatic cell type identification tool—R-package SingleR. For manual cell type annotation, we first applied the FindAllMarkers function in the R package Seurat to obtain the lists of genes for each cluster. Using prior knowledge of cell-type specific marker genes we subsequently assigned a cell type to each cluster (fibroblasts, smooth muscle cells (SMCs), endothelial cells, stem cells, etc.). The advantage of this method lies in its ability to define not only the highly expressed genes, which does not always imply their biological significance, but also the low expressed genes that may specify a certain cell type.

Cell cycle analysis was performed using the *CellCycleScoring* function of the Seurat R package. This algorithm classifies cells into distinct cell cycle phases by evaluating the expression of phase-specific marker genes. Curated gene sets corresponding to the G_2_/M and **S** phases were used to compute cell cycle scores based on the relative expression of these markers in each cell. The G_2_/M score reflects the expression of genes upregulated during the G_2_ and mitotic phases, whereas the S score represents genes involved in DNA synthesis. Cells were assigned to the G_1_, **S**, or G_2_/M phase according to the comparative analysis of these expression signatures. This approach enables precise characterization of cell cycle heterogeneity within single-cell datasets and supports downstream analyses that account for cell cycle–related variability in gene expression.

### 2.5 Adipogenic differentiation

MSCs isolated from healthy young donors were cultured in AdvanceSTEM Mesenchymal Stem Cell Media containing 10% AdvanceSTEM Supplement (HyClone, Cytiva, United States) and 1% antibiotic–antimycotic solution (HyClone, Cytiva, United States). For adipogenic induction, MSCs (five to seven passage) were seeded into a 12-well plate 2 days before each experiment and cultured until a confluent monolayer. Adipogenic medium comprised DMEM Low Glucose media (containing 1.0 g/L glucose) supplemented with L-glutamine (HyClone, Cytiva, United States), 10% fetal bovine serum (FBS) (HyClone, Cytiva, United States), containing 500 units/mL Antibiotic-Antimycotic (Gibco), and adipogenic induction factors (1 μM dexamethasone (Merck, Germany), 0.5 mM 3isobutyl-1-methylxanthine (IBMX, Millipore, United States), 10 μm/mL insulin (Paneco). For control conditions, cells were maintained in the medium without differentiation inducers. The culture media was replaced every 3 days.

### 2.6 Real-time live-cell monitoring of adipogenic differentiation in individual cells

MSCs (five to seven passage) were seeded into a 12-well plate 2 days before the experiment and cultured until a confluent monolayer. MSCs were transduced with lentiviral vectors (pLJM-Tcad–mKate for T-cadherin overexpression and pRPV-contr with the mKate as a control 2 days prior to adipogenic induction. These populations are referred to as T+vir and contr.vir, respectively. After transduction, cells were cultured either in adipogenic differentiation medium or in basal growth medium (control). Real-time live-cell monitoring was performed continuously for 10 consecutive days, with the adipogenic medium replaced every 3 days without interrupting of image acquisition.

Live cells were stained with Nile red (Sigma Aldrich (19123-10MG) for 40 min in Hank’s buffer (Paneco) stabilized with 50 mM HEPES (Paneco) pH 7.2 at 37 °C. Nile Red (Sigma Aldrich, 19123-10MG) at a concentration of 2 μg/mL was added to each well for 30 min at 37 °C in a 5% CO_2_ incubator. All wells were sequentially scanned using NIS-Elements (Nikon). Nile Red staining in living cells was imaged at (excitation/emission) 515/590 nm for total lipids (red) and 475/570 nm (emission) for non-polar lipids (green). The percentage of cells containing neutral lipids was analyzed using Nikon Eclipse Ti microscope equipped with a digital EMCCD camera Andor iXon 897 (Andor Technology, Belfast, United Kingdom). All wells were imaged in Whole well mode using a Nikon microscope [Nikon Eclipse Ti equipped with a digital EMCCD camera Andor iXon 897 (Andor Technology, Belfast, United Kingdom), NIS-Elements 5.21.02] with red (lentiviral transduction) and green (Nile Red) fluorescence registration. Cells at least in two wells per condition were analyzed in each experiment, and the experiment was repeated twice. The differentiation fate of each transduced cell was traced individually based on red fluorescence, with untransduced cells in the same culture serving as an internal control. Differences between characteristics were evaluated using Fisher’s exact test or Pearson’s chi-squared test and considered statistically significant at p < 0.05.

Next, cells were fixed, and T-cadherin was visualized using primary anti-T-cadherin antibody (ProSci, United States, #3583, dilution 1:100) and secondary Donkey anti-Rabbit IgG (H+L) Highly Cross-Adsorbed Secondary Antibody, AlexaFluor™ 350 Invitrogen A10039 (dilution 1:500). Images were analyzed using NIS-Elements (Nikon) and ImageJ software (NIH, Bethesda, MD, United States). Nile Red staining was imaged at (excitation/emission) 515/590 nm for total lipids (red) and 475/570 nm (emission) for non-polar lipids (green).

### 2.7 Oil red O and Nile red cell staining

The accumulation of intracellular lipid droplets upon differentiation was evaluated using Oil Red O or Nile Red staining followed by manual or automated analysis as previously described (Sysoeva et al., 2024).

Briefly, for Oil Red O staining, cells were fixed in 4% paraformaldehyde for 30–40 min at room temperature and rinsed with phosphate buffer saline (PBS). Oil Red O Solution (Sigma-Aldrich, United States) was added (500 μL–1 mL per well in a 24 well plate) for 50 min at room temperature. Oil Red O staining was visualized by Leica DMI6000B equipped with a digital camera Leica DFC7000T (Germany). Oil Red O staining was applied for illustrative representation of the results.

For Nile Red staining, the culture media was aspirated and replaced with warm Hank’s buffer (Paneco, Russia) supplemented with Hepes (Gibco) at a final concentration of 50 mM. Nile red (Sigma Aldrich, 19123-10MG) at a concentration of 2 μg/mL was added to each well with living cells for 30 min at 37 °C in a 5% CO_2_. All wells of the plate were sequentially scanned using NIS-Elements (Nikon). Nile Red staining was imaged at (excitation/emission) 515/590 nm for total lipids (red) and 475/570 nm (emission) for non-polar lipids (green). The percentage of differentiated cells was evaluated using AI-based image analysis as described below.

### 2.8 Automated quantification of adipogenic differentiation using AI-based image analysis

The ratio of differentiated adipocytes to total cells was quantified automatically using a custom algorithm developed with artificial intelligence and deep learning tools from the NIS.ai module of the *NIS Elements* software package (Nikon, v.5.42.02; https://www.microscope.healthcare.nikon.com/products/software/nis-elements/nis-ai-1). All cells in each experimental group were segmented based on the red fluorescence channel of Nile Red staining using the Segment.ai neural network from NIS.ai module of NIS Elements 5.42.02. The Segment.ai model processes an input image and produces a binary mask as output, segmenting and highlighting individual regions of interest allowing the segmentation of difficult-to-detect areas by learning on manually segmented dataset. It is capable of detecting complex structures by learning from manually annotated datasets. For this study, the model was fine-tuned on a training set of 20 manually labeled images (1,024 × 1,024 pixels) using the *Train Segment.ai* function, with 2,000 epochs and the *Dynamic Range Adaptation* option enabled. The resulting binary masks delineated individual cells, allowing accurate separation of adjacent cells and enabling quantitative assessment of cell counts and intracellular lipid content.

To determine the total number of cells, the *Object Count* function from the General Analysis 3 (https://www.microscope.healthcare.nikon.com/products/software/nis-elements/nis-ai-1, GA3) module was applied to the segmented cell masks. Lipid droplets were segmented by thresholding the red channel of Nile Red staining (the green channel was not used due to overlapping GFP expression in some cells). A manually defined threshold was applied consistently across all images within a given experiment. To identify cells containing lipid droplets, the *Having* function in GA3 was used to compare the binary masks of total cells and lipid droplets, generating a new mask representing only lipid-containing cells. These cells were quantified using the *Object Count* function in GA3. Finally, the differentiation ratio was calculated for each experimental group as the number of lipid-containing cells divided by the total number of cells.

### 2.9 Immunofluorescent analysis of adipose tissue

Subcutaneous adipose tissue was collected from 15 donors during abdominal surgery. All donors provided informed consent, and the study protocol was approved by the local ethics committee of the Medical Research and Education Center of Lomonosov Moscow State University (IRB00010587, Moscow, Russia) (#160, 22 July 2019, and #4, 4 June 2018, respectively). The average BMI of MSCs’ donors was 25.5 ± 3.1. Subcutaneous fat for MSC isolation was obtained from patients undergoing cosmetic abdominoplasty or elective surgeries, including surgeries for gallstones, infertility, pyelonephritis, and shoulder joint injury. The donors had no history of diabetes, cancer, or current symptoms of acute inflammation.

The tissue samples were embedded in O.C.T. Compound (Sakura Inc., Tokyo, Japan) and frozen in liquid nitrogen. Frozen sections of 15 μm thickness were prepared. The sections were fixed in 4% paraformaldehyde for 30 min. After washing with phosphate buffer saline (PBS), the sections were incubated in 0.1% bovine serum albumin (BSA) containing 10% normal donkey serum to block non-specific binding of antibodies. Subsequently, the sections were incubated with primary antibodies against DPP4 (CD26 Antibody, MA2607, ThermoFisher Scientific, dilution 1:100) and T-cadherin (ProSci, United States, #3583, dilution 1:100) for 1 h, followed by extensive washing in PBS. The visualization of DPP4 and T-cadherin staining was performed using secondary antibodies: Donkey anti-mouse IgG Highly Cross-Adsorbed Secondary Antibody, Alexa Fluor™ 594 (Invitrogen #A-21203 dilution 1:1,000), Donkey anti-Rabbit IgG Highly Cross-Adsorbed Secondary Antibody, Alexa Fluor™ 488 (Invitrogen #A-21206 dilution 1:1,000). Cell nuclei were counterstained with DAPI (Molecular Probes), and the sections were mounted in Aqua Poly/Mount (Polysciences Inc). Negative controls were used with appropriate concentrations of mouse or rabbit non-specific IgGs. Images were acquired by a confocal microscope LSM 780 and ZEN2010 software (Zeiss).

### 2.10 Immunofluorescent staining of MSCs

MSCs cultured on glass coverslips were fixed in 4% paraformaldehyde (PFA) for 10 min, washed in phosphate buffer saline (PBS) and incubated in PBS with 10% normal donkey serum. Cells were incubated with primary antibodies against DPP4 and T-cadherin, and secondary antibodies Donkey anti-mouse IgG Highly Cross-Adsorbed Secondary Antibody, Alexa Fluor™ 594, Donkey anti-Rabbit IgG Highly Cross-Adsorbed Secondary Antibody as described above. Cell nuclei were counterstained with DAPI and the glass coverslips were mounted in Aqua Poly/Mount. Images were acquired by a confocal microscope LSM 780 and ZEN2010 software (Zeiss) or Leica DMI 6000B equipped with Leica DFC7000T digital camera using LAS X software.

### 2.11 Proliferation assay using AI-based image analysis

MSCs (six passage), after lentiviral transduction with constructs for T-cadherin overexpression or control GFP vector and subsequent sorting with a cell sorter (LSR Fortessa flow cytometer, BD Biosciences; FACSDiva software, BD), were expanded to a subconfluent monolayer. In parallel, untransduced cells from the same donor were cultured under identical conditions. To evaluate proliferation rates, cells were seeded into 12-well plates at a density of 2 × 10^4^ cells/mL in 1 mL of growth medium, with four replicates per cell type. Phase-contrast images were captured every 24 h throughout the observation period, with the first acquisition performed 4 h after seeding. The medium was replaced every 4 days, and imaging was continued until a confluent monolayer was reached in at least one well. Cell proliferation was monitored over a 20-day period.

Phase-contrast images were obtained using a Nikon Eclipse Ti microscope equipped with a Nikon DS-Qi1Mc camera, Plan Fluor 10×/0.3 objective, and NIS Elements AR 5.40.02 software. The ND Acquisition module was used to record stage coordinates, ensuring that the same fields of view—and therefore the same cells—were imaged at each time point. Each field of view covered 36 mm^2^ (6 × 6 mm; 9,204 × 9,204 pixels per image).

Cell numbers in the time-lapse series were quantified using the *NuclePhaser* plugin for Napari developed by our lab (Voloshin et al., 2025). The YOLO-based model integrated into the plugin was trained to detect cell nuclei in phase-contrast images. For calibration, one representative image from each time series was manually annotated and adjusted using the “Calibrate with points” widget and the *Brightfield_v5l* model. The calibrated model was then applied to the image series with the “Predict on 1-stack” widget to obtain nuclei counts for each frame. The resulting data were aggregated and used to plot population growth graphs.

### 2.12 Flow cytometry

Cells were washed using Versen solution and incubated in HyQTase at 37 °C in a 5% CO_2_ incubator for 5–10 min. T-cadherin and DPP4 (CD26) expression in MSCs was analyzed using flow cytometry. Cells were detached from culture dishes using HyQTase Detachment Reagent (HyClone, GE Healthcare Life Sciences, United States) and stained with appropriate combinations of primary antibodies against: DPP4 (CD26 Antibody (MA2607), ThermoFisher Scientific, dilution 1:100), T-cadherin (ProSci, United States, #3583, dilution 1:100). Rabbit IgG Isotype Control (10500C, Invitrogen, 1:170) and normal mouse IgG (BD, 1:40) were used as negative controls. Stained live or fixed cells were washed with PBS and analyzed using a LSR Fortessa flow cytometer (BD Biosciences) and FACSDiva software (BD). The flow cytometry data were analyzed using FlowJo software (FLOWJO, LLC).

### 2.13 Cell lysates and Western blotting

MSC cells were plated in full growth medium onto 35 mm Petri dishes and were cultured up to 70%–80% confluence. HUVEСs were used as a positive control for T-cadherin expression. Cells were rinsed with cold PBS and scraped by rubber cell scraper (Corning) in lysis buffer (50 mM Tris-HCl, pH 8.0, 100 mM NaCl, 0.1 mM EDTA, 15 mM β-ME, 0.1 mM PMSF, 8% SDS, and 0.004% bromophenol blue and protease inhibitor cocktail 1:100). Samples were passed five times through a 30-gauge needle to splinter DNA. The loading amount was controlled by immunostaining for β-tubulin or GAPDH. Samples were electrophoresed in 10% SDS/polyacrylamide gel and electroblotted onto PVDF membrane (GE Healthcare). Kaleidoscope Prestained Standards (Bio-Rad Laboratories) were used as molecular weight markers. After rinsing in Tris-buffer saline (TBS: 150 mM NaCl, 50 mM Tris/HCl, pH 7.4), membranes were pre-blocked in TBSM buffer [TBS containing 5% (w/v) of delipidated milk and 0.5% Tween 20] for 120 min. Membranes were incubated with primary antibodies: anti T-cadherin (rabbit polyclonal, SantaCruz, sc-7940, dilution 1:500), anti- DPPIV/CD26 (mouse, Invitrogen #MA2607, dilution 1:1,500), anti-CEBP Beta (Affinity Biosciences, #AF0443, dilution 1:1,000), anti-GAPDH (SantaCruz, #sc-32233, dilution 1:1,000), anti-β-tubulin (SantaCruz, sc-9104, dilution 1:1,000) for 24 h at 4 °C, washed with TBSM, and then incubated with goat anti rabbit secondary antibodies conjugated with peroxidase (IMTEK, P-GAR Iss, dilution 1:3,000) for 1 h. Finally, membranes were washed in TBS containing 0.5% Tween 20 and visualized using SuperSignal West Pico Chemiluminescent Substrate (Thermo Scientific) and ChemiDoc™ XRS+ System (BioRad) for blot imaging and analysis.

### 2.14 Quantitative real-time polymerase chain reaction

Total RNA was extracted from human MSCs and quantified by a NanoDrop spectrophotometer. The following MSCs were enrolled into the study: control untreated cells, cells transduced with the construct for T-cadherin overexpression and cells transduced with control virus cultured either in the control or adipogenic medium for 10 days. The QuickRNA MicroPrep kit (#R1051, Zymo Research, United States) was applied according to the manufacturer’s instruction. The isolated RNA was treated with RNase-free DNAase I (Fermentas, Rockford, IL, United States) and then subjected to agarose gel electrophoresis for quality control. 1 µg of total RNA was reverse-transcribed to generate cDNA using the MMLV RT kit (Evrogen, Russia) and then subjected to real-time polymerase chain reaction (PCR) with qPCR mix-HS SYBR kit (Evrogen Russia) on a CFX96 Real-Time PCR Detection System (Bio-Rad). Primers were designed using NCBI Primer-BLAST: for DPP4 forward 5′-GIGACATCACTGCCACTGCCCACATC-3′ reverse 5′- GAA​TTA​TCC​GGT​CGG​TCG​GGT​TTT-3′; for RPLPO13 forward 5′-TCG​AAC​ACC​TGC​TGG​ACC​TGC​TGG​ATG​AC-3′, reverse 5′- GCA​CCA​TTG​AAA​TCG​AAA​TCC​TGA​GTG​A-3′; for T-CADHERIN forward 5′-CTG​TCA​CTA​TCG​CAC​TAT​CGA​CTA​CCT​TGC-3′, reverse 5′- GCA​AGA​TAT​GCT​ATG​GCA​GAA​CTC​GTG-3′.

The thermal cycling program for template denaturation, primer annealing, and primer extension was 40 cycles of 94 °C for 15 s, 60 °C–62 °C for 30 s, and 72 °C for 15 s, respectively. The relative transcript level of mRNA was calculated using the 2^−ΔΔCT^ method with *RPLPO13* as a reference; normalization was done assuming as one the mean level of each transcript in control group (control).

### 2.15 Statistical analysis

For each adipose tissue section obtained from a donor, 10 random fields of view were photographed, and the percentage of T-cadherin+ and DPP4^+^ positive cells, as well as double-positive cells, was calculated. Statistical analysis was performed using Prism software (GraphPad Software, La Jolla, California, United States). The Kolmogorov-Smirnov test and Shapiro–Wilk test were used to check for normal distribution of the data. For the normally distributed data, the unpaired Student’s t-test was applied to assess significant differences between the groups, whereas for non-normally distributed data, the Mann–Whitney rank-sum test (Wilcoxon rank-sum test) was used. Differences with a p-value of <0.05 were considered statistically significant.

For RT-qPCR, data were analyzed using GraphPad Prism 9 software (GraphPad Software Inc., United States). All data were assessed for normal distribution using the D’Agostino-Pearson normality test. Data are presented as the mean + standard deviation (SD). Differences in gene expression were determined using one-way analysis of variance (ANOVA) following Dunnett *post hoc* test for multiple comparisons, Unpaired T-test was used to compare data when there were two groups.

For real-time live-cell monitoring of adipogenic differentiation in individual cells, the differences were evaluated using Fisher’s exact test or Pearson’s chi-squared test and considered statistically significant at p < 0.05.

## 3 Results

### 3.1 Identification of DPP4^+^ cells in the population of T-cadherin expressing cells in human subcutaneous adipose tissue

Merrick and co-authors identified a population of DPP4^+^ interstitial progenitors that undergo commitment to the adipose lineage, thereby facilitating the overall cell renewal of adipose tissue (Merrick et al., 2019). Mature adipocytes of various types display distinct transcriptional profiles and secrete different adipokines (Bäckdahl et al., 2021), yet the mechanisms by which adipokines regulate adipocyte differentiation remain poorly understood. Of note, among mature adipocytes only one subtype (Adipo^PLIN^) expresses adiponectin (Bäckdahl et al., 2021) and T-cadherin operates as the exclusive receptor for its high-molecular-weight (HMW) form, which is the most metabolically active. Despite this, the spatial distribution of T-cadherin within adipose tissue has not been mapped, nor has its expression been examined in DPP4^+^ progenitor cells.

**FIGURE 1:**
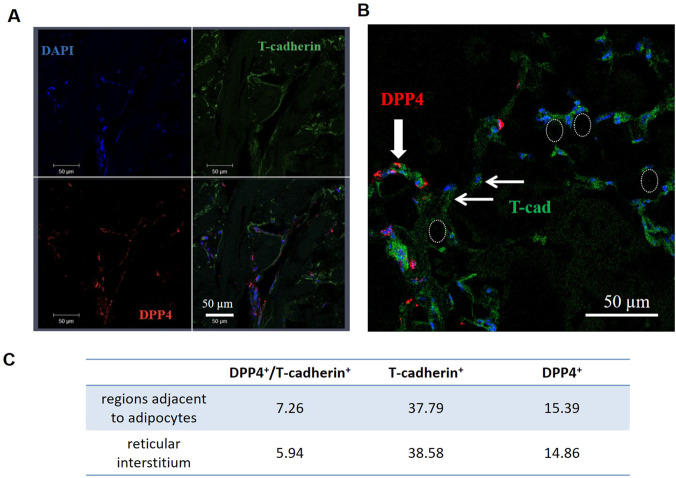
Double immunofluorescent staining of human subcutaneous adipose tissue sections with antibodies against T-cadherin (green) and DPP4 (red); nuclei were counterstained with DAPI (blue). Images were acquired using a Zeiss LSM 780 confocal microscope and ZEN2010 software, shown at lower magnification **(A)** and higher magnification **(B)**. A thick arrow points to a group of cells expressing both T-cadherin and DPP4 in the interstitium; thin arrows mark cells expressing only T-cadherin; ovals encircle adipocytes. Scale bar 50 µm. **(C)** The table shows the percentage of T-cadherin–positive, DPP4^+^ cells and double-positive cells (DPP4^+^/T-cadherin^+^), quantified from adipose tissue sections of two healthy donors.

To investigate the spatial distribution of these cell populations, we performed double immunofluorescent staining for DPP4 and T-cadherin on sections of subcutaneous adipose tissue from healthy donors (Figure 1). T-cadherin was broadly expressed in adipose tissue: T-cadherin-positive cells were located adjacent to blood vessels or surrounded large adipocytes (Figures 1A,B). DPP4^+^ cells showed a similar distribution within the adipose tissue adipocytes (Figures 1A,B). Quantitative analysis revealed that only a small fraction of DPP4^+^ cells co-expressed T-cadherin, accounting for approximately 1% of the total cell population (Figure 1C). Although the abundance of double-positive (DPP4^+^/T-cadherin^+^) cells varied among donors, no statistically significant differences were detected between their occurrence in the reticular interstitium and in regions adjacent to unilocular adipocytes.

### 3.2 Identification of DPP4^+^ cells in the subpopulation of T-cadherin expressing mesenchymal stem cells (MSCs) *in vitro*

**FIGURE 2:**
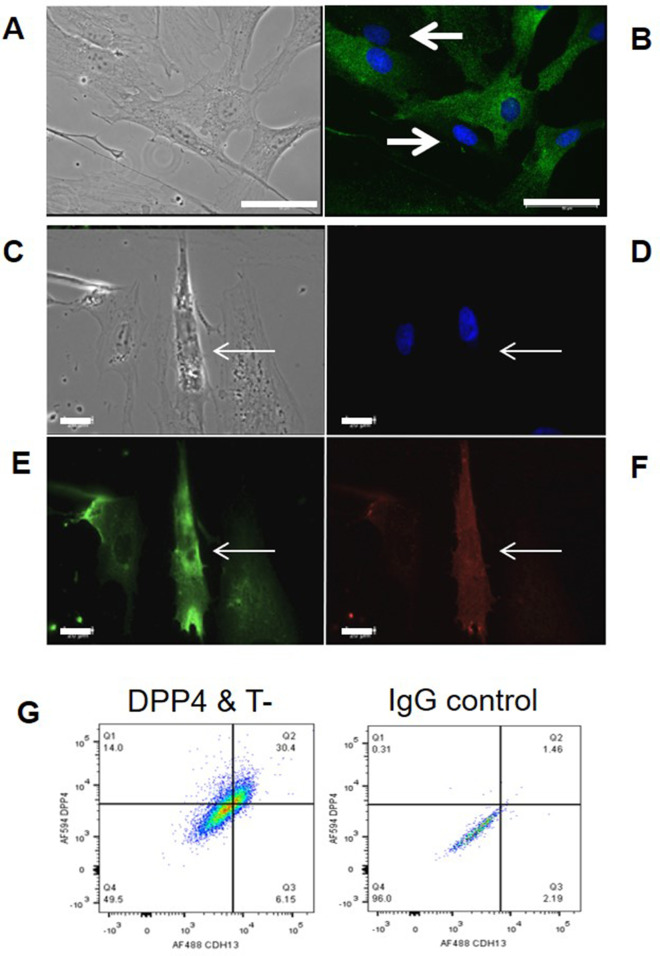
Light microscopy of MSCs (of the two to three passages) isolated from human subcutaneous adipose tissue of a healthy donor **(A)** and immunofluorescent staining with antibodies against T-cadherin (green) **(B)**. Arrows indicate cells with low or no T-cadherin expression, whereas cells exhibiting green fluorescence corresponding to T-cadherin are clearly visible. Scale bar, 50 µm. Light microscopy of human MSCs **(C)** and double immunofluorescent staining with antibodies against T-cadherin green, **(E)** and DPP4 red, **(F)** nuclei were counterstained with DAPI blue, **(D)**. Arrows in **(C–F)** indicate one and the same cell co-expressing T-cadherin and DPP4. Images were acquired using a Leica DMI 6000B microscope equipped with a Leica DFC7000T digital camera and LAS X software. Scale bar, 20 µm. **(G)** Representative flow cytometry plot showing T-cadherin and DPP4 distribution in cultured MSCs. The proportion of double-positive (DPP4^+^/T-cadherin^+^) cells was 30.4%; 6.15% expressed only T-cadherin, and 14% expressed only DPP4.

Having identified DPP4 and T-cadherin expression in human adipose tissue we addressed a question of whether this expression pattern is retained in cultured mesenchymal stem cells (MSCs). MSCs of the two to three passages were obtained from healthy donors and stained with antibodies against T-cadherin. Immunofluorescent analysis revealed a mosaic T-cadherin expression containing both T-cadherin-positive and T-cadherin-negative cells (Figures 2A,B). Double immunofluorescent staining of MSCs revealed that a subset of T-cadherin–positive cells also expressed DPP4 up to the fourth passage (Figures 2C–F). Quantitative evaluation of MSCs isolated from subcutaneous adipose tissue of five healthy donors using flow cytometry analysis revealed that T-cadherin–positive cells accounted for 15%–38% of all cell in culture, with donor-dependent variability. In early passages, approximately 10%–15% of these cells co-expressed DPP4, and most DPP4^+^ cells were also positive for T-cadherin (Figure 2G).

### 3.3 Induction of adipogenic differentiation results in a significant decline in the number of T-cadherin expressing MSCs

**FIGURE 3:**
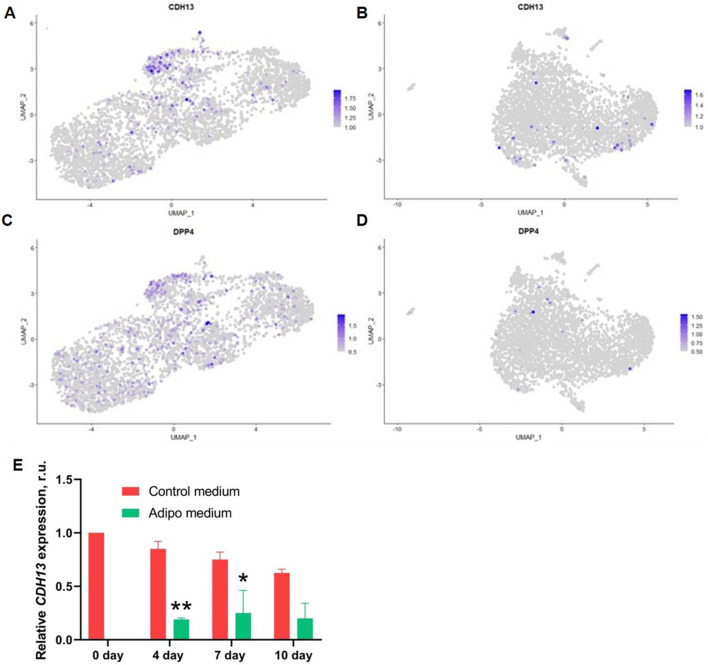
Individual UMAP plots showing the expression levels and distribution of *CDH13* (encoding T-cadherin) in control MSCs **(A)** and MSCs after 4 days of adipogenic induction **(B)**. UMAP plots demonstrating *DPP4* expression in control MSCs **(C)** and MSCs after 4 days of adipogenic induction **(D)**. **(E)** RT-qPCR analysis of MSCs cultured in control medium or under adipogenic induction conditions showing the dynamics of T-cadherin mRNA expression. T-cadherin/*CDH13* expression decreased by day 4 in adipogenic medium and remained low through day 10. RT-qPCR data are shown as the mean ± SD. T-test. **р< 0.01 *p < 0.05 vs. control media in corresponding experimental day. Results are representative of three biologically independent experiments.

Recently, single-cell mRNA sequencing (scRNA-seq) has been applied to generate largely unbiased and substantially more comprehensive insights compared to earlier approaches (Burl et al., 2018; Maniyadath et al., 2023). To explore the role of T-cadherin in the structure of adipogenic stem and precursor cell subpopulations, we performed scRNA-seq on human MSCs cultured in control conditions or 4 days after induction of adipogenic differentiation. First, we found that in the control undifferentiated sample (3,459 cells) the percentage of T-cadherin expressing cells was 59%, while in the sample of cells committed to adipogenic lineage (3,435 cells) it decreased up to 34%. Therefore, the 4-day adipogenic induction led to a drastic decline in the number of T-cadherin-expressing cells (by a factor of 1.7) (Figures 3A,B).

To further verify the bioinformatic findings, we performed quantitative RT-PCR on MSCs isolated from the subcutaneous adipose tissue from two healthy donors (Figure 3E). Early-passage MSCs were seeded in Petri dishes and grown to confluence. Next, the cells were cultured in control medium or in medium containing the adipogenic factor cocktail. The culture medium was changed every 2–3 days. On days 4, 7, and 10 the total RNA was isolated, and T-cadherin expression level was assessed. Upon adipogenic induction on day 4, T-cadherin mRNA expression in the MSCs decreased by almost 5-fold compared to cells cultured in control medium, and remained at this level up to 10 days (Figure 3C). Therefore, both bioinformatic and RT-qPCR analysis showed that the proportion of T-cadherin-positive cells significantly decreased upon adipogenic differentiation. These results indicate that, although T-cadherin is generally downregulated under adipogenic conditions, a subpopulation of cells among heterogeneous MSCs retains T-cadherin expression even during adipogenic induction.

Merrick and co-authors (Merrick et al., 2019) proposed a developmental hierarchy for human adipose stem and progenitor cells, showing that these populations undergo lineage commitment in response to adipogenic stimuli. Within this hierarchy, they identified DPP4^+^ cells as a renewable source of preadipocytes and mature adipocytes. In parallel, T-cadherin has been recognized as a specific and physiologically relevant receptor for HMW adiponectin, the form exhibiting the highest metabolic activity (Rubina et al., 2021; Hug et al., 2004; Hara et al., 2006; Ma et al., 2013). Based on this, we hypothesized that T-cadherin may regulate adipogenic differentiation, potentially through an adiponectin-mediated feedback loop. T-cadherin may be involved in regulating the adipogenic differentiation or may well be maintaining the compartment of stem/progenitor cell renewal within adipose tissue. We hypothesized, and subsequently demonstrated, that T-cadherin is expressed in progenitor MSCs, with the proportion of these cells declining over time in culture or following induction of adipogenic differentiation. To address this question in detail, we performed scRNA-seq analysis.

### 3.4 Identification of *CDH13* expressing subpopulation of MSCs concurrently expressing stemness genes and DPP4 as a marker of interstitial progenitors in the adipose tissue

**FIGURE 4:**
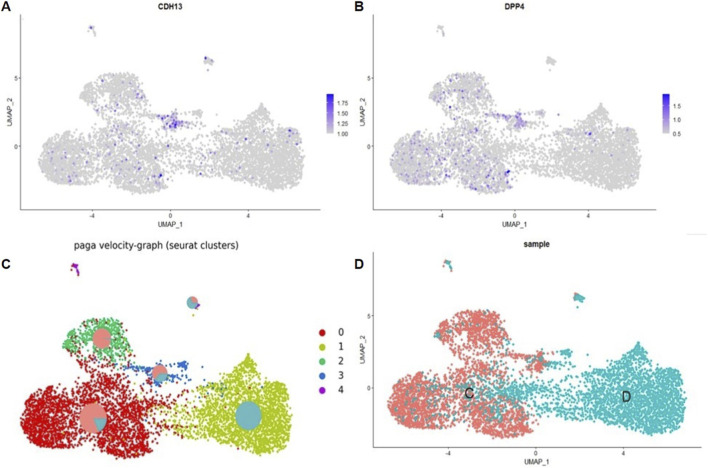
Integrated object. **(A)** FeaturePlot–UMAP-plot showing principal distribution of *CDH13* gene expression (encoding for T-cadherin) in the integrated object; *CDH13* expressing cells corresponds to Cluster 3 (more than 1-fold change of the average expression level); **(B)** FeaturePlot–UMAP-plot showing principal distribution of *DPP4* gene expression (encoding for T-cadherin) in the integrated object; *DPP4* expressing cells correspond to Cluster 3 (more than 1-fold change of the average expression level) **(C)** DimPlot–Integrated object UMAP-clustering. Sample proportion diagrams depict the ratio between the cell counts in the control MSC sample (Salmon) and in the MSC sample (Iris blue) after a 4-day induction of adipogenic differentiation within the Clusters. **(D)** DimPlot–Integrated object grouped by samples. *CDH13* expression in the control MSC sample (Salmon) and MSC sample (Iris blue) after a 4-day induction of adipogenic differentiation. Cluster 3 predominantly contains cells from the control sample.

**FIGURE 5:**
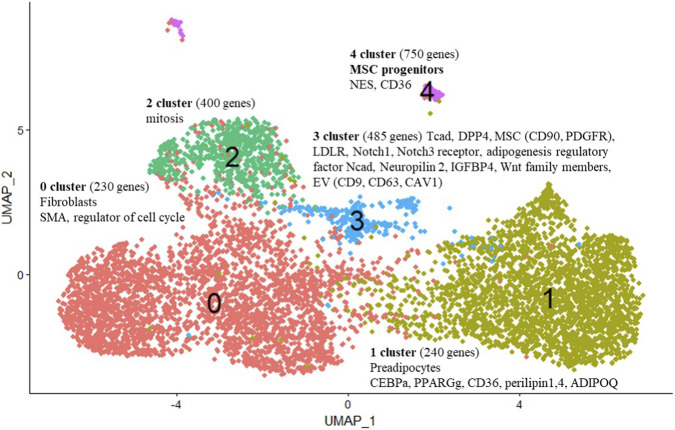
Integrated object. FeaturePlot. Each cluster is denoted by color. Cluster 0 (Salmon) primarily contains cells expressing fibroblast markers and genes responsible for cell cycle regulation. Cluster 1 (Khaki) encompasses cells expressing preadipocyte-specific genes, such as *CEBPB*, PPARγ, *CD36* and markers of mature adipocytes (*ADIPOQ*, *Perilipin1*, *Perilipin4*). In Cluster 2 (green), cells predominantly express genes related to mitosis. Cluster 3 (Blue) contains cells of interest with high level of T-cadherin expression, as well as classical MSC markers (*CD90*, *PDGFR*), *Wnt signaling genes*, and *DPP4*. In a separate remote Cluster 4 (Magenta), besides *CDH13*, cells express *Nestin*, a marker of neural crest cells, and *CD36*, a marker of adipocyte progenitors.

We bioinformatically analyzed the representation of genes in human MSC subpopulations, comparing cells maintained under control conditions with those subjected to adipogenic induction, across different stages of the adipogenic hierarchy in relation to T-cadherin expression. We found that *CDH13*/T-cadherin–expressing cells (Figures 3A,B) largely overlapped with DPP4-expressing cells (Figures 3C,D), a marker of interstitial progenitor cells specifically implicated in *de novo* adipogenesis (Merrick et al., 2019). We next integrated the scRNA-seq data, obtained from the control MSCs and MSCs after a 4-day induction of adipogenic differentiation (Figure 4). After clustering, we found that cells with a high level of *CDH13* (Figure 4A) were predominantly located in Cluster 3 (Figures 4A, 5). We integrated the obtained scRNA-seq data (Figure 4C), and clustering revealed that cells with high *CDH13* expression predominantly corresponded to the cells of the control sample (Figures 3A,B, 4A,D).

We performed the cell type annotation of all clusters, using automatic cell type identification tool and manually assigning a cell type to each cluster (Figure 5). For manual cell type annotation, we applied the list of genes generated based on the prior knowledge of cell-type specific marker genes (Supplementary Material S1: genes of adipogenic differentiation are highlighted in yellow; stemness-related genes are in red). We carried out the cell type assigning of fibroblasts, smooth muscle cells (SMCs), endothelial cells, stem cells and progenitors, preadipocytes, etc., to each cluster (Figure 5). We revealed that, in Cluster 3, in addition to *CDH13*, the cells expressed stemness-related genes such as *DPP4*, *Notch1*, *Notch3 receptor*, key MSC markers (*CD90*, *PDGFR*), *Wnt* family members, *LDLR*, adipogenesis regulatory factor *CDH2*, *Neuropilin 2*, *IGFBP4*, and genes involved in exosome biogenesis (*CD9*, *CD63*, *CAV1*) (totally 485 genes) (Figures 4A,B, 5).

**FIGURE 6:**
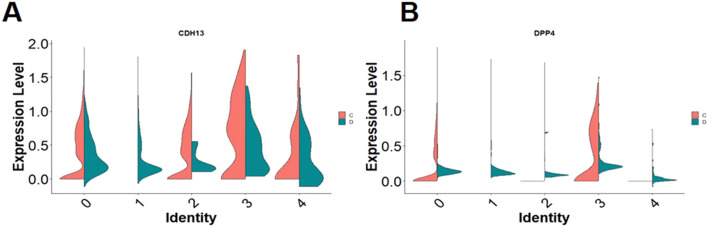
Split violin-plots showing the relative expression levels and distribution of *CDH13* **(A)** and *DPP4* **(B)** genes in the control MSC sample (Salmon) and MSC sample after a 4-day induction of adipogenic differentiation (Iris blue). The highest *CDH13* expression was detected in Cluster 3 in MSCs of the control sample compared to MSCs after a 4-day adipogenic induction. Similarly, the highest expression of *DPP4* was found in Cluster 3 in MSCs of the control sample. Split violin plots were generated using the R package Seurat and the function VlnPlot with the argument split.by = “sample”.

Of note, Cluster 3 was specifically enriched with the cells expressing *DPP4* (Figures 4B, 6). Split violin plots demonstrated that the highest *CDH13* gene expression was detected in Cluster 3, specifically in MSCs in the control sample compared to MSCs after adipogenic induction in terms of both, the range of gene expression (vertical height) and the number of cells (horizontal width from the baseline to the edge of the curve) (Figure 6A). In line with this, the same pattern of gene expression in Cluster 3 was obtained for *DPP4* (Figure 6). The parallel distribution of *CDH13* and *DPP4* suggests that these MSC subpopulations (*CDH13* ^*+*^ and *DPP4* ^*+*^) may be interrelated and overlapping.

The heterogeneity of MSCs may arise from multiple factors, including donor-related variables (age, sex, health status, genetic background) and methodological differences in MSC isolation and expansion (choice of digestion enzyme, matrix protein, culture medium, passage number, etc.) (Chen et al., 2024). To validate our findings, we repeated all clustering and bioinformatic analyses using publicly available datasets. For this purpose, several candidate datasets were evaluated. At the initial stage, three datasets were considered. The first was series GSE129363, available in the GEO (Gene Expression Omnibus) database of the National Center for Biotechnology Information (NCBI). This dataset was of particular interest because of its methodological design and the comprehensive data it provides. The second source was the large-scale Tabula Sapiens project (version 1.0), hosted in the Figshare repository under accession number 14267219 (https://figshare.com/articles/dataset/Tabula_Sapiens_release_1_0/14267219). This resource was notable as a comprehensive atlas of human single-cell gene expression. The third candidate was series GSE182158, also accessible via the NCBI GEO platform. Following a detailed comparative assessment of technical parameters, data quality, and relevance to our research objectives, we selected series GSE182158 as the most suitable dataset, owing to its optimal sequencing quality, coverage depth, and experimental design. This series included 11 biological samples, of which three (GSM5519464, GSM5519465, and GSM5519466) were chosen as the most representative based on cell yield, sequencing depth, and library quality. After preliminary quality assessment of these three samples, GSM5519464 was selected for subsequent analysis, as it also comprised experimental data directly relevant to our study.

Dataset processing was performed using the R package Seurat v5.0.2. Filtering criteria were set as follows: *nFeature_RNA* > 1,500 and *nFeature_RNA* < 5,000, *nCount_RNA* > 5,000 and *nCount_RNA* < 35,000, and *percent.mt* < 5. The first 30 principal components were analyzed. Clustering was performed with the *FindClusters* function at a resolution of 0.3, and cluster annotation was carried out manually. Cell type identification using the *FindAllMarkers* function was conducted with the parameters *only.pos = FALSE*, *min.pct = 0.05*, and *logfc.threshold = 0.05*.

**FIGURE 7:**
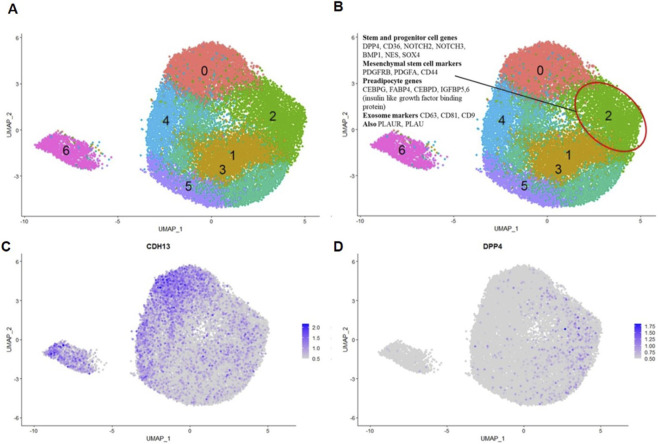
**(A)** DimPlot–GSE182158 object UMAP-clustering; **(B)** 2 cluster manual cell type annotation, the red oval marks cluster 2; **(C)** FeaturePlot–UMAP-plot showing principal distribution of *CDH13* gene expression in the GSE182158 object; **(D)** FeaturePlot–UMAP-plot showing principal distribution of *DPP4* gene expression (encoding for T-cadherin) in the GSE182158 object.

The clustering results confirmed our observations, demonstrating the co-expression of *DPP4* and *CDH13* in Cluster 2 of the control sample (Figure 7). This suggests the presence of a distinct subpopulation within the heterogeneous MSC pool that simultaneously expresses both markers. Detailed gene analysis of this cluster revealed, in addition to *DPP4*, the expression of multiple stem and progenitor cell–related genes (*CD36*, *NOTCH2*, *NOTCH3*, *BMP1*, *NES*, *SOX4*). These cells also displayed mesenchymal stem cell markers (*PDGFRB*, *PDGFA*, *CD44*), exosomal protein genes (*CD63*, *CD81*, *CD9*), as well as preadipocyte-associated genes (*CEBPG*, *FABP4*, *CEBPD*, *IGFBP5*, *IGFBP6*, *PLAUR*, *PLAU*, *TGFBR3*) (Figure 7). Thus, these findings demonstrate that cells co-expressing *DPP4* and *CDH13* are present not only in our analyzed samples but also across a range of publicly available datasets. More detailed data on gene expression in cells from publicly available datasets are presented in the Supplementary Material S2.

### 3.5 Cell cycle profiling of the integrated sample

**FIGURE 8:**
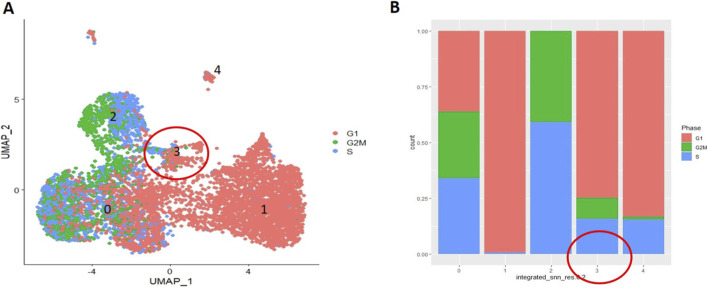
Integrated object UMAP-clustering. **(A)** Cell clustering based on cell cycle phase–associated gene expression. **(B)** Proportion of cells in each cell cycle phase across clusters 0–4. Phases are color-coded, and Cluster 3 is marked with a red circle.

Progenitor and stem cells are known to differ from the bulk cell population in certain aspects of cell cycle regulation (Singh et al., 2019; Liu et al., 2019; Mohammad et al., 2019). To further characterize Cluster 3 cells co-expressing *CDH13* and *DPP4*, considered as potential progenitors, we analyzed their cell cycle phase distribution across clusters using the *CellCycleScoring* function (Figure 8).

To assess the proliferative status of cells in each cluster, we determined the proportion of cells in the G1, S, and G2/M phases in both control and adipogenically induced samples. We also examined cell cycle distribution across all clusters of the integrated dataset, with particular attention to those expressing *CDH13*. In Cluster 0 (fibroblasts and SMA^+^ cells), cells were evenly distributed across all phases. In Cluster 1, annotated as preadipocytes, nearly all cells were in G1. By contrast, Cluster 2 contained no cells in G1, and most of its highly expressed genes were associated with mitotic processes. In Cluster 3, previously annotated as enriched for cells expressing *CDH13*, *DPP4*, exosome markers, and transcription factors—approximately 75% of the cells were in G1, 10% in G2/M (actively proliferating), and 15% in S phase. A comparable distribution was observed in Cluster 4, although the proportion of proliferating cells was substantially lower, consistent with the expression of mesenchymal progenitor markers such as *nestin* and *CD36* in this cluster. Taken together, the *CDH13* ^*+*^ cells in Cluster 3 were predominantly non-proliferative, consistent either with a dormant state, a hallmark of stemness in certain stem cell populations (Singh et al., 2019; Liu et al., 2019; Mohammad et al., 2019), or with a differentiating state characteristic of preadipocytes (Fajas, 2003), which we explored in more detail below.

### 3.6 T-cadherin overexpression results in augmented DPP4 expression and decreased proliferative capacity of human MSCs *in vitro*

**FIGURE 9:**
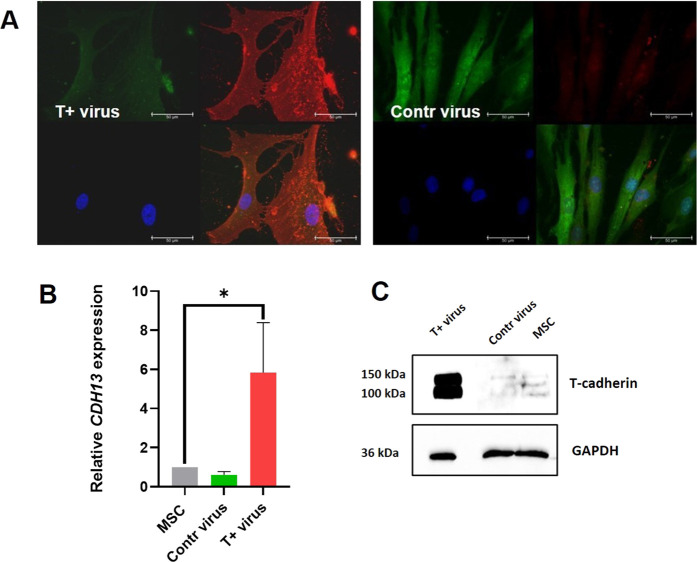
T-cadherin overexpression in human MSCs using lentiviral constructs comprising GFP for cell identification and sorting. Overexpression of T-cadherin in human MSCs using lentiviral constructs carrying either GFP alone (control) or T-cadherin–GFP, allowing subsequent cell identification and sorting. **(A)** T-cadherin overexpression (T+virus) was confirmed using immunofluorescent staining with antibodies against T-cadherin (red) and compared to Control virus cells (Contr virus). Cell nuclei were counterstained with DAPI (blue). Scale bar 50 m. T-cadherin overexpression was verified applying RT-qPCR **(B)** and Western blot **(C)**. GAPDH was used as the loading control **(C)**. Results from two biologically independent RT-qPCR and eight Western blot experiments are shown.

To underpin our hypothesis on the *CDH13* and DPP4 expression being tightly intertwined, we transduced human MSCs with lentiviral vectors for T-cadherin overexpression or the control virus (both with the fluorescent tag GFP). Further, using FACS, cells were sorted based on GFP fluorescence, and T-cadherin overexpression was verified using the immunofluorescent staining with antibodies against T-cadherin, RT-qPCR, and Western blot. RT-qPCR analysis indicated that T-cadherin level in T-cadherin overexpressing cells exceeded both controls by more than 7-fold (Figure 9). Microscopy and Western blot analysis further confirmed the RT-qPCR results (Figure 9).

**FIGURE 10:**
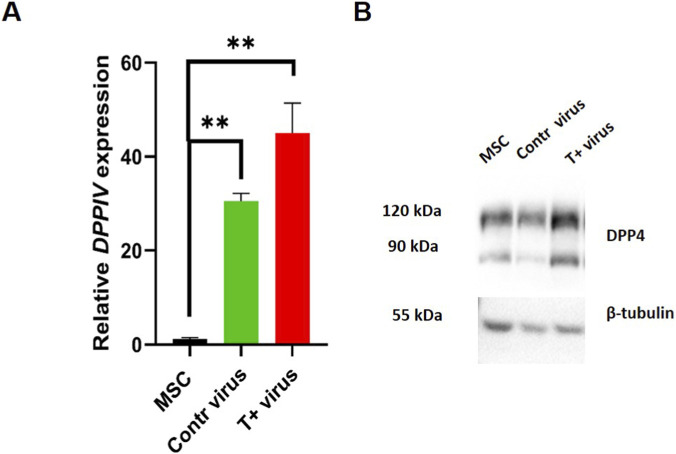
Elevated DPP4 expression in MSCs after lentiviral transduction in T-cadherin-overexpressing cells was verified using RT-qPCR **(A)** and Western blot **(B)**. β-tubulin was used as the loading control for Western blot analysis. Representative results from one of two biologically independent RT-qPCR and eight Western blot experiments are shown. ANOVA with multiple comparisons, **p < 0.01.

We sought to test DPP4 expression (Figure 10) as a marker of adipocyte progenitors in relation to T-cadherin level of expression. Using RT-qPCR and Western blot we examined DPP4 expression in MSCs after lentiviral transduction for T-cadherin overexpression and compared with two controls (cells transduced with the control virus or untransduced cells from the same donor). We revealed that T-cadherin overexpression significantly increased the DPP4 expression both at the mRNA and protein levels (Figure 10).

It has been previously reported that lentiviral GFP transduction can, in some cases, alter the expression of target genes or sensitize cells to various stimuli. For instance, GFP expression has been shown to increase oxidative stress and enhance the sensitivity of neuroblastoma cells to cytotoxic drugs (Goto et al., 2003), as well as to affect cellular gene expression in other systems (Liu et al., 1999). Therefore, we enrolled two types of control cells in our experiments: untrunsduced cells and cells transduced with the control GFP lentivirus. While our results revealed a clear upregulation of DPP4 in T-cadherin–overexpressing cells compared with both controls, a slight increase in DPP4 expression was also observed following GFP lentiviral transduction alone. Of note, previous studies have demonstrated that GFP transduction does not adversely affect the cellular, biochemical, or phenotypic characteristics of human MSCs (Yu et al., 2015). Therefore, we considered comparison with GFP-transduced cells to be the most appropriate control for assessing the specific effects of T-cadherin overexpression on DPP4 expression.

**FIGURE 11:**
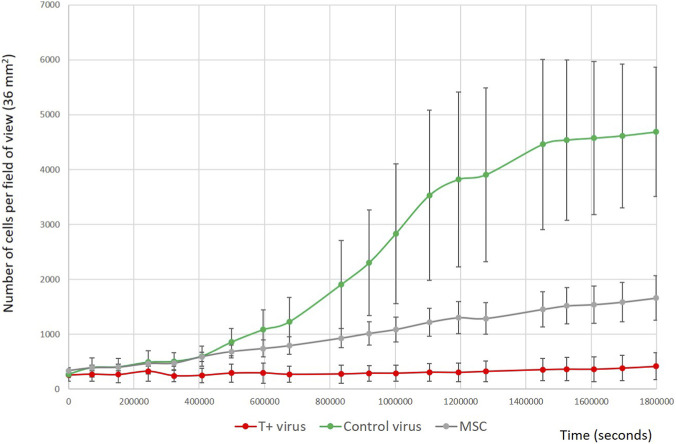
T-cadherin–overexpressing MSCs demonstrated reduced proliferative capacity compared to control cells (either transduced with a control virus or untransduced donor-matched MSCs).

Next, we examined the effect of T-cadherin overexpression on MSC proliferation (Figure 11). Cell numbers were quantified as described in the Materials and Methods section using a neural network–based approach that enabled accurate detection and counting of individual cells at each time point. This method has been previously described in detail by our group (Chechekhina et al., 2024). MSCs with different levels of T-cadherin expression exhibited markedly distinct proliferation dynamics. T-cadherin–overexpressing cells showed minimal proliferative activity, maintaining a nearly constant cell number throughout the experiment. In contrast, control cells (both those transduced with a control virus and untransduced donor-matched MSCs) displayed normal population growth (Figure 11).

To infer, clustering of the integrated dataset demonstrated that MSCs with elevated *CDH13* expression (≥1.5-fold) and those with high *DPP4 e*xpression localized within the same cluster (Cluster 3). Detailed gene analysis of this cluster revealed, in addition to *DPP4*, the expression of multiple stem and progenitor cell–associated genes and mesenchymal stem cell markers. Cells expressing T-cadherin/*CDH13* exhibited low proliferative activity, with approximately 75% of the cells being in the G_1_ phase. Of note, the proportion of *CDH13*-expressing cells declined, following adipogenic induction.

In line with this, T-cadherin overexpressing cells demonstrated substantially reduced proliferation rate. In this regard, we hypothesized that MSCs with a high level of T-cadherin/*CDH13* simultaneously expressing high *DPP4* may represent a dormant stem-like subpopulation in the adipose tissue. Quiescent or dormant subpopulations of stem cells with a reduced proliferative potential have already been acknowledged in various organs and tissues (Sottocornola and Lo Celso, 2012).

### 3.7 T-cadherin expression correlates with the presence of stemness-related genes in human MSCs

We next examined the expression pattern of other stemness-related genes under control conditions or upon adipogenic differentiation in relation to T-cadherin/*CDH13* in MSCs. To this end, we generated heatmaps using the Loupe Browser visualization tool (v. 6.4.1) from 10x Genomics. For that the output. cloupe file from the CellRanger count pipeline was used followed by application of cellranger aggr to obtain the.aggr file from samples of control MSCs and the cells after a 4-day adipogenic induction. Since Cell Ranger’s algorithm makes its own clustering, the results may differ from the clustering performed when applying the barcodes.tsv, features.tsv, and matrix.mtx files in R package Seurat. Specifically, Seurat integration yielded 5 clusters, while Cell Ranger processing produced 11 clusters. Loupe Browser allows to rename clusters, therefore in the aggregated sample along with the corresponding numbers, we additionally labeled the clusters belonging to the control cluster with letter “C” and clusters belonging to the sample after induction of adipogenic differentiation with letter “D”. Upon cluster visualization, the cluster names were ranked sequentially and in an alphabetic order allowing for the differences in the aggregated samples to appear. A list of genes of interest prepared in advance was loaded into Loupe Browser via the Active Feature List tab under the Gene/Feature Expression category. Two separate datasets were generated: cells with high T-cadherin expression (more than 1-fold change of the average expression level in a sample) and cells with low T-cadherin (less than 1-fold change). For each dataset, heatmaps were generated for stemness genes. For that we applied the Filters function and Assign Selection. Subsequently, Heat Map tool was selected.

**FIGURE 12:**
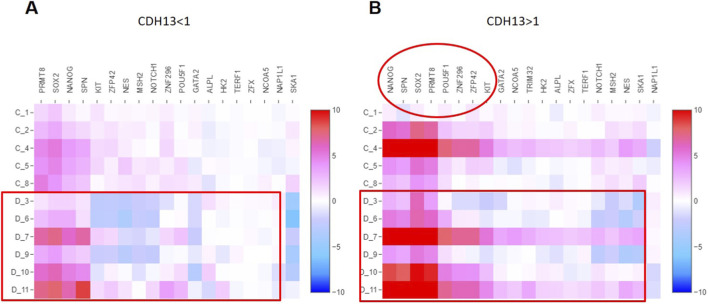
Heatmap of stemness-related genes in clusters of the control and adipocyte-induced MSC samples. MSCs with low level of *CDH13* expression (less than 1-fold change of the average expression level in the sample) **(A)** MSCs with high *CDH13* expression level (more than 1-fold) **(B)**. Clusters related to the control sample are labeled as C1-C8; clusters of differentiating cells are labeled as D3-D11, respectively.

First and foremost, the level of several stemness-related genes (*NANOG*, *SPN*, *SOX2*, *PRMT8*) in cells with high *CDH13* was elevated at the background of the cells with low *CDH13* expression (Figure 12). Moreover, expression of these stemness-related genes was maintained even upon adipogenic induction (clusters C compared to clusters D). Interestingly, in some clusters in Figure 12B the expression levels of all stemness-related genes were significantly higher both in the control (C4) and differentiating cells (D7, D11) correlating with high *CDH13* expression. More detailed data on gene expression in cells from these clusters are presented in the Supplementary Material S1.

Our heatmap-based approach along with other findings, demonstrating a clear relationship between the level of T-cadherin expression and stemness-related markers, allowed us to assume that T-cadherin plays an essential role in preserving MSCs from adipogenic differentiation, maintaining their stem-like status.

### 3.8 T-cadherin expression in human MSCs inhibits adipogenic differentiation *in vitro*

To gain further insight into the role of T-cadherin in regulating adipogenic differentiation we set out to test the functional properties and adipogenic potential of cells, transduced with the construct for T-cadherin overexpression or the control lentivirus (referred to as T+vir culture and contr.vir culture) (Figure 14).

**FIGURE 13:**
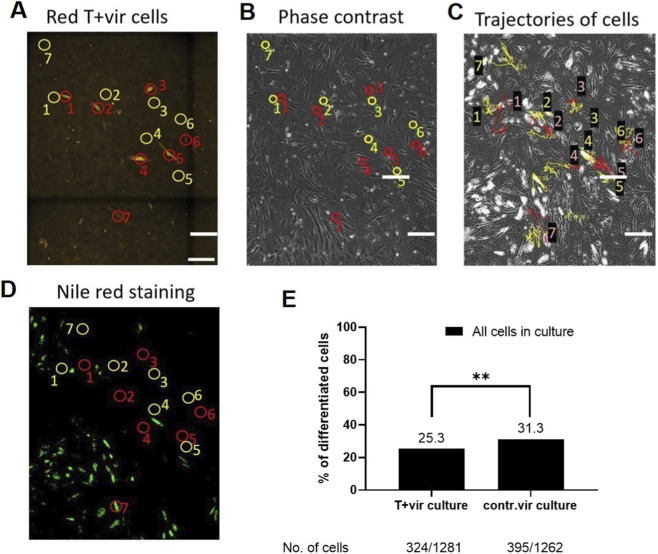
Human MSC culture after lentivirus transduction for T-cadherin overexpression (representative images for the cell culture transduced with lentivirus for T-cadherin overexpression). Images of MSCs were captured through a long-term live-cell imaging over a period of 10 days. Transduced cells were identified by red fluorescence (mKate fluorescence with emission maximum 633 nm, pseudo-colored in yellow) **(A)**. Cells displaying mKate fluorescence and overexpressing T-cadherin are encircled in red, while the neighboring untransduced cells serve as internal control and are encircled in yellow **(A,B)**. Panels **(C,D)** show the same field of view as in **(A,B)** after 10 days [**(C)**—phase contrast; **(D)** green fluorescence channel]. Nile Red staining was used for visualization of non-polar (neutral) lipids (green fluorescence channel) **(D)**. Images were acquired using a microscope NIS-Elements (Nikon) and ImageJ software (NIH, Bethesda, MD, United States). Scale bar 100 μm. **(E)** Quantification of cells undergoing adipogenic differentiation in MSC cultures transduced with lentivirus for T-cadherin overexpression or the control virus (T+vir culture and contr.vir culture). **p = 0.0008.

For registration of adipogenic differentiation on individual cells in real-time, we plated T+vir and contr.vir cells in the standard culture media immediately after transduction and 2 days later induced them towards adipogenic differentiation. The differentiation fate of each transduced cell was then traced individually based on red fluorescence (mKate as a label for transduced cells); the untransduced cells in the same well served as an internal control. The long-term monitoring of the cells in real-time was performed for 10 consecutive days. In total, 506 cells in T+vir-culture and 472 cells in contr.vir culture were analyzed. The efficiency of viral transduction in the T+vir culture was 4.7% (24 red cells out of 506 cells examined) and 6.6% (31 red cells out of 472 cells) in the contr.vir culture. The individual cells and their differentiation fate were monitored based on: the mKate fluorescence, accumulation of intracellular lipid droplets with their visualization using Nile Red, and anti-T-cadherin staining with antibodies for confocal microscopy at the end of the experiment (Figure 13; Supplementary Video).

Quantitative analysis of the proportions of differentiated and undifferentiated cells in T+vir and contr.vir cultures revealed that T-cadherin overexpressing MSCs remained in an undifferentiated state and did not accumulate lipid droplets, as confirmed by individual cell fate tracking combined with Nile Red staining (Figure 13; Supplementary Video). Tracking the fate of individual cells with red fluorescence in the T+vir cultures (T-cadherin overexpressing cells marked by red circles) and comparing them with the neighboring untransduced cells within the same wells (marked by yellow circles) (Figures 13A–D) clearly demonstrated that T-cadherin overexpressing cells were resistant to adipogenic differentiation, as most of these cells exhibited no detectable Nile Red staining (Figure 13D).

**FIGURE 14:**
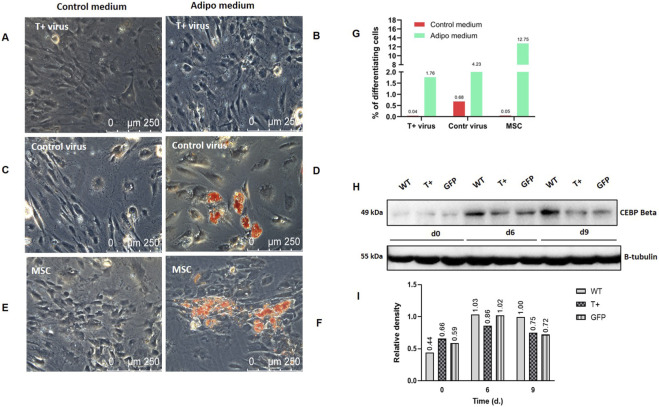
Adipogenic differentiation in MSC cultures consisting of untransduced cells, cells transduced with either lentivirus for T-cadherin overexpression or control virus, following 14 days of culture in either control or adipogenic media. **(A–F)** Bright field and phase-contrast micrographs of MSC cultures after Oil Red O staining. Distinct orange lipid droplets are visible in individual cells and cell clusters in untransduced MSCs, and control virus–transduced MSCs, whereas T-cadherin–overexpressing MSCs show no detectable Oil Red O staining. Representative images from one of two biologically independent experiments are shown. Scale bar 250 μm. **(G)** Quantification of differentiated cells following Nile Red staining using AI-based image analysis and deep learning algorithms from the NIS.ai module, showing the ratio of differentiated adipocytes to total cells. **(H)** Western blot assessment of an early adipocyte differentiation marker CEBP Beta in lysates of MSCs [WT—untransduced cells, cells transduced with either lentivirus for T-cadherin overexpression (T+) or control virus (GFP)] cultured in adipogenic or control condition for 9 days. For loading control, β-tubulin antibody was used. (d0 – start of the experiment; d6, d9—days following adipogenic induction). **(I)** Densitometric quantification of Western blot analysis of CEBP Beta levels.

To further validate our hypothesis that T-cadherin overexpression reduces the adipogenic differentiation potential of MSCs, we sorted the cells following viral transduction based on their fluorescence, and induced adipogenic differentiation. After 14 days in adipogenic medium, cells in selected wells were stained with Nile Red, and the proportion of differentiated cells was quantified using AI-based image analysis (Figure 14G). In parallel, cells in the neighboring wells were stained with Oil Red O and examined using a Leica microscope in combined bright-field and phase-contrast (Bf–Ph) mode (Figures 14A–F). The graph in Figure 14G demonstrates that after 14 days of culturing in adipogenic medium, MSCs overexpressing T-cadherin accumulated lipid droplets significantly less efficiently (1.76%) than control cells (4.23% for MSCs transduced with control virus and 12.75% for donor-matched MSCs without viral transduction).

Representative micrographs of Oil Red O-stained cells in Figures 14A–F illustrate lipid accumulation and corroborate the AI-based image quantification results of Nile Red staining. For optimal visualization, cells were imaged using combined bright-field and phase-contrast microscopy. In control wells containing MSCs cultured with adipogenic inducers (D, F), individual cells and small clusters with Oil Red O-stained lipid droplets were clearly visible. Under the same conditions, T-cadherin overexpressing MSCs did not accumulate lipid droplets (B). In control medium (A, C, E), cells of all three types exhibited minimal lipid accumulation, consistent with the AI-based quantification of Nile Red staining results (Figure 14G; red column). Western blot analysis of an early adipogenic marker CEBP Beta in MSC lysates revealed that T-cadherin-overexpressing cells exhibited reduced CEBP Beta levels compared to both untransduced cells and those transduced with the control lentivirus on days 6 and 9 (Figures 14H, I).

### 3.9 Gene profiling and trajectory analysis of human MSCs using pseudotemporal trajectories from scRNA-seq

**FIGURE 15:**
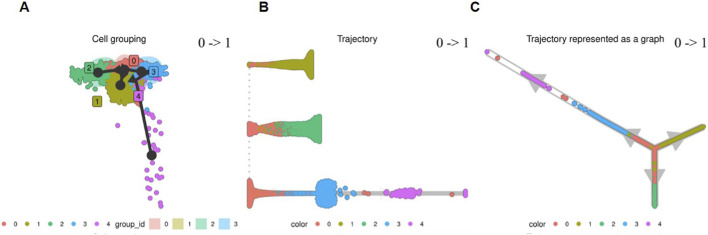
Developmental trajectory analysis of MSCs transcriptional states in the integrated object. **(A)** Slingshot-based analysis of the cell fate in the integrated object was performed using two approaches presented in **(B,C)**. **(B)** 0->1 Cluster 0 identified as fibroblasts and specified as the origin, and Cluster 1 identified as preadipocytes and specified as the endpoint. **(C)** Three branching events were detected: first, from Cluster 0 to Cluster 1 (preadipocytes); second, from Cluster 0 to Cluster 2 (cells expressing mitotic genes); third, from Cluster 0 to cluster 4 (cells expressing NESTIN and MSC progenitor markers).

We applied Slingshot to construct the pseudotemporal trajectories from scRNA-seq and examine the role of T-cadherin in adipogenic differentiation, specifically in stem cells and early progenitors, pre-adipocytes and differentiated adipocytes (Figure 15). Among the well-known trajectory construction methods, Slingshot is considered to be one the most accurate one (Saelens et al., 2019; Street et al., 2018). We initiated Slingshot data processing by performing clustering in a low-dimensional space. A minimum spanning tree was then constructed based on these clusters to infer lineage trajectories. Next, preliminary lineage paths passing through the clusters were refined into smooth continuous curves representing distinct lineages. Finally, pseudotime values were assigned to each cell by computing its orthogonal projection onto the corresponding trajectory curve. We explored the potential cellular transition path from Cluster 0, predominantly composed of undifferentiated cells from the control sample, to Cluster 1, which contained the majority of differentiated cells (Figure 15). Automatic cell type identification revealed that, Cluster 0 was primarily consisted of cells identified by their gene expression profiles as fibroblasts and smooth muscle cells and cells expressing cell cycle regulatory genes (230 genes). In contrast, Cluster 1 mostly comprised cells of the differentiated sample expressing genes of early and late adipogenic markers, such as *CEBPα*, *PPARGγ*, *CD36*, *perilipin1* and *4*, *ADIPOQ* (240 genes). A primary objective was to determine whether cells from Cluster 3 were involved in the transition from Cluster 0 to Cluster 1. Based on the transcriptional profiles, we identified three trajectory directions representing distinct transitions of transcriptional states for the majority of undifferentiated cells grouped in Cluster 0 (Figure 15).

- Cells from Cluster 0 (pink) appeared to transit directly to Cluster 1 (2,672 cells) (khaki), the latter exhibiting gene expression profile of preadipocytes.
- Cells from Cluster 0 (pink) transitioned directly to Cluster 2 (699 cells) (green), the latter exhibiting genes related to mitosis (Figure A, middle panel, middle trajectory).
- Cells from Cluster 0 (pink) have transited through Cluster 3 (333 cells) (blue), where cells express *CDH13* and *DPP4*, and subsequently redistributed to Cluster 4 (90 cells) (magenta), characterized by the expression of *CD36*, an early marker of adipocyte progenitors with a high degree of differentiation capacity (Gao et al., 2017), and *NESTIN*, a marker of stem cells and progenitors of neural and mesenchymal lineages (Bernal and Arranz, 2018). Cells from this spatially separated Cluster 4 are presumably descendants of neural crest cell lineage primed for adipogenic differentiation (Figure 5).

Based on the generated tree of transcriptional states, we defined a possible scenario, by which cells from Cluster 3 enact in transition between cell types. In this scenario, actively cycling cells from Cluster 0 can redistribute to Cluster 3 of early progenitors expressing *CDH13* and *DPP4*. Subsequently Cluster 3 cells can contribute to Cluster 4, where cells express other markers of pluripotency and stemness-related genes, such as *NESTIN*, *CD36*, *KIT*, *SCA-1*, *PDGFR*, *SOX4*, *NOTCH3*, etc. Since the transition of cells from Cluster 0 (actively cycling cells) to Cluster 1 (preadipocytes and committed adipocytes) does not involve Cluster 3, we concluded that Cluster 3 cells with high *CDH13* and *DPP4* expression occupy a distinct position among other cell clusters and are not engaged in adipogenic differentiation, at least at the early stages following adipogenic induction.

## 4 Discussion

The heterogeneity in the field of adipose tissue cells, as well as of MSCs derived from WAT, has been a long-standing question that reflects the intrinsic complexity of adipose tissue organization and renewal regulation. This cellular diversity underlies the functional specialization of distinct progenitor subpopulations, while also representing a major challenge to elucidating the molecular mechanisms that govern lineage commitment and adipogenic differentiation (Maniyadath et al., 2023; Chen et al., 2024; Horwitz et al., 2005).

Bäckdahl et al., performed spatial mapping of human subcutaneous WAT and identified 18 distinct cell types, which were annotated to mature adipocytes, adipocyte progenitors, vascular and immune cells. Studies using integrative analyses of transcriptional profiling of human WAT composition (Bäckdahl et al., 2021), as well as single-cell RNA sequencing of isolated MSCs detected functionally distinct cell populations contributing to the ongoing discussion within the cell hierarchy in the adipose tissue (Vishvanath and Gupta, 2019; Merrick et al., 2019; Schwalie et al., 2018; Bäckdahl et al., 2021; Hatzmann et al., 2022; Min et al., 2019). Based on the marker genes, Bäckdahl et al., identified three types of mature adipocytes: Adipo^LEP^, Adipo^PLIN^, and Adipo^SAA^ enriched in genes encoding specific adipokines—leptin, perilipin, and adiponectin, respectively (Bäckdahl et al., 2021). More recently, single nucleus RNA sequencing (snRNA-seq) identified seven distinct subpopulations of mature adipocytes (hAd1–hAd7) in human subcutaneous WAT(51). Adipocyte subpopulations displaying similar marker expression profiles, gene programs, functional characteristics, and enrichment in related signaling pathways across studies can be grouped as: hAd3/4 Adipo^PLIN^ adipocytes exhibiting lipogenic activity associated with lipid metabolism and insulin signaling; hAd3/Adipo^SAA^ cells involved in lipid and retinol handling and inflammatory responses; hAd2/4/5/Adipo^LEP^ adipocytes linked to leptin signaling, stress response, and extracellular matrix (ECM) remodeling; hAd1/7 cells defined by enrichment in pathways related to calcium signaling and cell–ECM interactions (Bäckdahl et al., 2021; Maniyadath et al., 2023; Emont et al., 2022). Collectively, these findings utilizing scRNA-seq and snRNA-seq analysis demonstrated that WAT comprises several functionally distinct adipocyte subpopulations, with some being more lipogenic while others specialized in lipid uptake and metabolism (Maniyadath et al., 2023). The diversity of adipocyte subtypes and their functions, along with the repertoire of secreted adipokines, may be attributed to distinct origins of adipocytes arising from specific progenitors within WAT, thus highlighting that adipocyte heterogeneity may reflect the diversity among their progenitors.

We hypothesized that T-cadherin, being a specific receptor for HMW adiponectin (Fukuda et al., 2021) may mediate a feedback loop and operate as a regulator in the compartment of naive stem/progenitor cells, controlling their proliferation and adipogenic differentiation. Recently, we explored the role of T-cadherin in adipogenic differentiation, as well as the effects of its well-known ligands, LDL and adiponectin on this process using murine MSCs. We confirmed that these ligands exert distinct and context-dependent effects. While LDL promoted adipogenic differentiation, T-cadherin expression attenuated LDL-induced lipid accumulation, suggesting a modulatory role for T-cadherin. In contrast, LMW adiponectin suppressed lipid droplet accumulation, but this effect appeared to be largely independent of T-cadherin. Conversely, the absence of T-cadherin sensitized MSCs to the anti-adipogenic effects of HMW adiponectin, indicating that T-cadherin may act as a regulatory hub, modulating cellular responsiveness to metabolically active ligands, LDL and adiponectin, and their signaling during adipogenesis (Sysoeva et al., 2024).

Within the framework of cell fate continuum in the adipose tissue, Merrick et al. positioned DPP4^+^ cells at the pinnacle of the adipogenic hierarchy, ascribing DPP4 as a marker to multipotent early progenitors that serve as a renewable source of preadipocytes and give rise to ICAM1^+^ (enriched in *Pparg* expression) and CD142^+^ preadipocytes, which are subsequently committed to differentiate into mature adipocytes (Merrick et al., 2019). Using scRNA-seq and cell trajectory analyses, Merrick et al., identified DPP4^+^ cells as highly proliferative progenitors that are relatively resistant to adipogenic differentiation. Unlike committed preadipocytes that reside in the perivascular region within WAT, Merrick et al., revealed that more primitive DPP4^+^ cells occupy an anatomically distinct compartment surrounding the adipose tissue, a specific niche referred to as the reticular interstitium (Merrick et al., 2019). However, other studies, reported DPP4^+^ cells in close proximity to the adipose vasculature within inguinal (iWAT) and gonadal (gWAT) depots (Schwalie et al., 2018; Rondini and Granneman, 2020). Collectively, these findings indicate that adipose progenitor cells can be distributed across multiple anatomical niches, spanning both perivascular and interstitial regions within adipose depots (Maniyadath et al., 2023). To establish the spatial expression patterns of T-cadherin and DPP4, we first explored their distribution using sections of human subcutaneous adipose tissue. T-cadherin was broadly expressed in adipose tissue, with T-cadherin–positive cells located both within or adjacent to blood vessels and around large adipocytes. Of note, DPP4^+^ cells exhibited a similar spatial expression pattern. DPP4/T-cadherin double-positive cells were found in close proximity to large adipocytes and within the reticular interstitium in accordance with the previously published results (Merrick et al., 2019). Quantitative analysis revealed that only a small fraction of DPP4^+^ cells co-expressed T-cadherin with no statistically significant differences in their abundance between the reticular interstitium and regions adjacent to unilocular adipocytes (Figures 1A–C). Of note, the proportion of double-positive (DPP4^+^/T-cadherin^+^) cells varied among donors. The majority of isolated and cultured MSCs retained T-cadherin expression during the early passages in basal growth medium (Figure 2G), while adipogenic stimuli led to a marked decrease in T-cadherin expression, as demonstrated by UMAP-plots and RT-qPCR results (Figure 3). Approximately 10%–15% of the early-passage cells concurrently expressed DPP4 in culture, and most of these DPP4^+^ cells also co-expressed T-cadherin *in vitro* (Figure 2G).

Functionally, DPP4^+^ cells express typical MSC genes (Hatzmann et al., 2022), canonical markers of somatic stemness (KLF4, c-MYC) and quiescence markers (p21^Cip1^, p27^Kip1^, p57^Kip2^) (Hatzmann et al., 2021), while lacking pluripotency markers (NANOG, SOX2, OCT4) (Hatzmann et al., 2022). The loss- and gain-of-function experiments by Hatzmann et al., supported the concept that DPP4 expression sustains proliferation and stemness. The knockdown of DPP4 suppressed the proliferative and self-renewal capacity of MSCs while enhancing adipogenenic differentiation potential, whereas DPP4 overexpression significantly decreased adipogenesis. These findings indicated that DPP4 is a marker of MSCs with high stemness, playing a crucial role in preserving the stem cell phenotype and undifferentiated state in human MSCs (Hatzmann et al., 2022). Beyond being expressed in progenitors, DPP4 performs multiple regulatory functions in mature adipocytes, where its expression increases during differentiation and impacts insulin signaling (Röhrborn et al., 2016; Lamers et al., 2011). The release of soluble DPP4 increases during adipogenic differentiation and reaches its highest levels in mature adipocytes (Rosmaninho-Salgado et al., 2012). These findings suggest that DPP4 plays an important role in adipogenic differentiation and that adipose tissue is a significant source of circulating DPP4.

Elevated DPP4 levels in adipose tissue and circulation correlate with obesity and insulin resistance, implicating soluble DPP4 (sDPP4) in paracrine regulation of metabolic and inflammatory processes as an adipokine (Sell et al., 2013). Mechanistically, DPP4, a membrane-bound and soluble peptidase, modulates the local microenvironment by processing chemokines, growth factors, and incretins such as GLP-1. Thereby DPP4 affects not only glucose metabolism, but also key aspects of progenitor cell biology, such as viability, mobilization, and immune regulation (Zhao et al., 2014; Ou et al., 2013; Barchetta et al., 2022). Several of these substrates are components of the mesenchymal stem cell (MSC) niche, suggesting that incretins and other DPP4-regulated peptides may affect multiple physiological aspects of MSC behavior (Torrecillas-Baena et al., 2022). Although the direct effects of incretins on MSCs are not yet fully understood, emerging evidence indicates that GLP-1 upregulates MSC proliferation while diminishing apoptosis. At the same time, GLP-1 inhibits adipogenesis by downregulating C/EBPβ and PPARγ expression and promotes osteogenic differentiation (Torrecillas-Baena et al., 2022; Sanz et al., 2010). Given that DPP4 expression in MSCs can modulate the local availability of incretins, it is plausible that DPP4 acts as a molecular link between systemic metabolic signals and local stem cell behavior in the adipose tissue. Collectively, these findings support a model in which DPP4^+^ serves as a marker of early self-renewing progenitors/stem-like cells that sustain adipose tissue homeostasis, while the DPP4 protein itself functions as a metabolic modulator across different stages of adipocyte lineage commitment and differentiation (Hatzmann et al., 2022).

To investigate the distribution of T-cadherin/*CDH13* expression and its potential functional role across MSC subpopulations cultured under control conditions and after 4 days of adipogenic induction we performed scRNA-seq. Following the integration and clustering of scRNA-seq data, we observed a notable co-occurrence of high *CDH13* and *DPP4* expression levels within Cluster 3, primarily aligning with cells from the control sample (Figures 3–6). We performed automatic and manual cell type annotation of all clusters and revealed that, cells in Cluster 3 displayed typical MSCs gene expression profiles (*CD90*, *PDGFR*, and etc. (Merrick et al., 2019)) (Figure 5, Supplementary Material S1). However, besides the overlapping *CDH13* and *DPP4* expression, Cluster 3 cells exhibited high levels of stemness-related genes such as *Notch1* and *Notch3 receptor*, *Wnt family members*, *N-cadherin*, *Neuropilin 2*, *IGFBP4*, *LDLR*, *adipogenesis regulatory factor*, and etc. (totally 485 genes) (Figure 5).

We further generated heatmaps using the Loupe Browser, and separated cells with high and low T-cadherin expression within the clusters of control and differentiated MSC samples (Figure 12). Surprisingly, cells with high T-cadherin/*CDH13* expression exhibited elevated levels of several stemness-associated genes (*NANOG*, *SPN*, *SOX2*, *PRMT8*, but not *OCT4*) in the control sample, and these levels remained high even after 4 days of adipogenic induction, suggesting a potential link between *CDH13* expression and the maintenance of a stem-like transcriptional state. Therefore, our findings are only partly in line with the earlier published study by Hatzmann et al. (Hatzmann et al., 2022), which demonstrated that none of the canonical pluripotency markers (*NANOG*, *SOX2* or *OCT4*) were substantially expressed in either DPP4^+^ nor in DPP4^−^ MSCs, as tested directly following cell sorting.

To explore the functional interconnection between T-cadherin and DPP4, we overexpressed T-cadherin in human MSCs using lentiviral construct (Figure 9). Remarkably, T-cadherin overexpression resulted in the elevated DPP4 mRNA and protein levels as shown by RT-qPCR and Western blot (Figure 10), highlighting a potentially important regulatory link whereby T-cadherin may act upstream of DPP4 to influence the proliferation and adipogenic differentiation of DPP4^+^ progenitor cells. Next, we performed cell tracing to monitor the fate and adipogenic differentiation of individual cells in real time using long-term live-cell imaging over a 10-day period, complemented by Nile red staining of live cells and Oil red O cell staining of fixed cells. As demonstrated in Supplementary Video, Figures 13, 14, T-cadherin overexpression drastically abrogated MSCs adipogenic potential. These findings are in line with our previously published results on murine MSCs and well-established 3T3-L1 mouse preadipocyte cell line, where forced expression of T-cadherin similarly suppressed adipogenesis in both cell models, while T-cadherin suppression resulted in spontaneous differentiation into adipocytes with the formation of large lipid droplets (Sysoeva et al., 2024; Klimovich et al., 2025). Interestingly, in the present study, we found that T-cadherin overexpression, in addition to suppressing adipogenic differentiation, also downregulated MSC proliferation, suggesting that T-cadherin–positive cells may represent a dormant MSC subpopulation with stem-like properties and intrinsically low adipogenic differentiation potential (Figures 8, 11). Of note, previous reports described DPP4^+^ MSCs as highly proliferative progenitors that similarly exhibit a limited adipogenic capacity (Merrick et al., 2019; Kyung et al., 2024). Moreover, while DPP4 overexpression was shown to dramatical abrogate adipogenesis, DPP4 knockdown reduced proliferation and self-renewal while enhancing adipogenic differentiation (Merrick et al., 2019; Hatzmann et al., 2022; Kyung et al., 2024). Our data only partially align with these findings and support a model in which T-cadherin, potentially acting upstream of DPP4, maintains MSC stemness within a small subpopulation of cells by attenuating both proliferation and adipogenic commitment. Together, these observations highlight the functional heterogeneity among early MSC subsets and point to a finely tuned regulatory axis between T-cadherin and DPP4 in defining progenitor fate.

Finally, by employing Slingshot, we generated pseudotemporal trajectories from the scRNA-seq data. This analysis enabled us to outline a potential scenario for the involvement of T-cadherin-expressing cells in transitions between different cell types (Figure 15). The method applied supports the concept that T-cadherin expressing cells constitute a discrete cell subpopulation characterized by stem-like properties, and they are not involved directly in adipogenic differentiation. Consistent with that paradigm, our pseudotemporal trajectory analysis placed the T-cadherin^high^/DPP4^+^ cells on a separate branch removed from the main adipogenic differentiation path. Rather than progressing toward adipocyte development, this subpopulation retains a stem-like transcriptional profile, suggesting that T-cadherin marks cells inclined to remain as quiescent progenitors rather than active adipocyte precursors. These findings support the notion that T-cadherin marks a discrete MSC subpopulation with stem-like or regulatory properties and are consistent with our *in vitro* results, reinforcing the conclusion that T-cadherin being upstream of DPP4 overexpression restrains adipogenic differentiation potential.

Taken together our findings suggest additional heterogeneity within the DPP4^+^ progenitor population. While ultimately having a low adipogenic potential, DPP4^+^ population can be further subdivided into a highly proliferative subpopulation as described earlier (Merrick et al., 2019; Hatzmann et al., 2022) and a subpopulation of DPP4^+^ cell that simultaneously express T-cadherin. The latter is characterized by the expression of stemness genes, low adipogenic potential, and limited proliferation capacity.

Our findings change the conceptual landscape model of T-cadherin functions and introduce additional layers of complexity to the adipose tissue’s hierarchy. Initially recognized as a member of cadherin cell adhesion family and as a receptor for LDL and HMW adiponectin (Rubina et al., 2021), T-cadherin has unexpectedly emerged as an important regulator within the compartment of naive stem/progenitor cells. Its role involves regulating the proliferation and adipogenic differentiation of a subset of early progenitors, thereby potentially shaping the composition, consistency, and functional diversity of mature adipocytes and emerging adipocyte subtypes within adipose tissue. T-cadherin may act as an upstream regulatory checkpoint within the adipose progenitor hierarchy, potentially upregulating DPP4 as part of a transcriptional program associated with stemness. However, DPP4 expression in this context may not reflect immediate functional activation, especially if adipogenic signaling pathways are repressed by T-cadherin. Rather, DPP4 may serve as a marker of a primed, quiescent progenitor state, in which progression towards proliferation and differentiation is restrained by other T-cadherin–mediated mechanisms—such as adhesion, cytoskeletal dynamics, or adiponectin signaling—with T-cadherin maintaining cells in an undifferentiated condition until specific activation cues emerge. Thus, the T-cadherin-DPP4 axis may define an intermediate progenitor population, primed for lineage commitment but restrained from full differentiation.

Further in-depth studies using *in vitro* and *in vivo* models are needed to unravel the precise mechanisms through which T-cadherin exerts its effects in the adipose tissue, including the role of its ligands (LDL and HMW adiponectin) in shaping the T-cadherin-positive subpopulation, and to identify potential signaling partners and/or microRNA encoded within the *CDH13* gene. In addition, exploring how T-cadherin–expressing MSCs respond under different metabolic conditions, such as those mimicking obesity or insulin resistance, will be critical for understanding their physiological and pathophysiological relevance. The important agenda for future research will also involve clarifying the role of T-cadherin in both health and disease, particularly its potential involvement in linking the elevated DPP4 expression in adipose tissue to obesity and metabolic syndrome.

## Data Availability

The dataset used in this study can be accessed publicly at: https://www.ebi.ac.uk/ena/browser/view/PRJEB102099.
