# Supplementary material for: Adiponectin receptor T-cadherin emerges as a novel regulator of adipose stem cell quiescence and adipogenesis
Source: Front Cell Dev Biol. 2026 Jan 15;13:1734183. doi: 10.3389/fcell.2025.1734183 (PMC12851984; doi:10.3389/fcell.2025.1734183)
Supplement: Supplementary file 1 [file Table2.docx]

**Supplementary material**

To further validate our scRNA-seq data on the co-expression of *DPP4* and *CDH13* within the same clusters of human MSCs, we analyzed publicly available datasets, although resources suitable for in-depth examination were limited. We selected the GSE129363 dataset from the GEO database (NCBI, which contains subcutaneous adipose tissue samples from 11 obese individuals (GSM3711761, GSM3711762, GSM3711765, GSM3711766, GSM3711769, GSM3711770, GSM3711772, GSM3711774, GSM3711777, GSM3711778, GSM3711781) (<https://www.ncbi.nlm.nih.gov/geo/query/acc.cgi?acc=GSE129363>; doi: [10.1038/s42255-019-0152-6](https://doi.org/10.1038%2Fs42255-019-0152-6)).

We performed dataset analysis using the R package Seurat v5.0.2. Filtering was conducted using the parameters nFeature_RNA > 200 & nFeature_RNA < 1000 & nCount_RNA > 200 & nCount_RNA < 5000 & percent.mt < 20. The first 30 components were analyzed. The FindClusters function was executed to obtain clusters with the parameter resolution = 0.3. Cell type annotation was performed using automatic cell type identification tool. The FindAllMarkers function for marker identification was run with parameters only.pos = FALSE, min.pct = 0.05, logfc.threshold = 0.05.

Cell type annotation was performed using automatic cell type identification tool (Blueprint DB) (Figure S1). We identified co-expression of *DPP4* and *CDH13* in Clusters 1 and 2, which were annotated as fibroblasts, adipocytes, smooth muscle cells, and (DOI: 10.1038/s42255-019-0152-6), our analysis confirmed the presence of progenitor cells co-expressing ***CDH13* and** *DPP4* in Clusters 1 and 2 (Figure S1).

Additionally, we detected ***CDH13*** and *DPP4* expression in endothelial cells within Cluster 6, consistent with the existing literature data (doi: [10.3389/fendo.2019.00080](https://doi.org/10.3389%2Ffendo.2019.00080); doi: 10.1007/s004180100252).

|  |  |
| --- | --- |


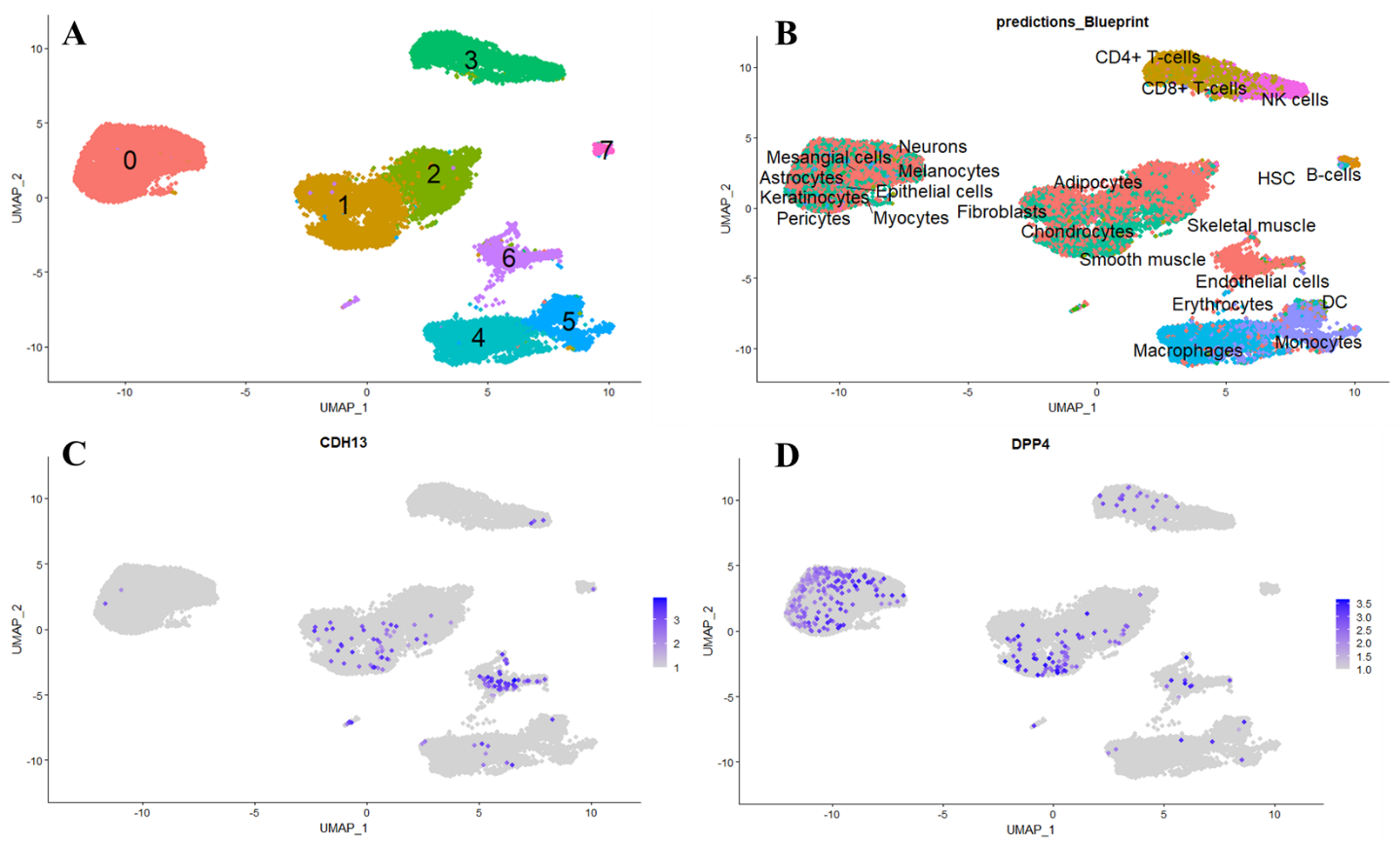


**Figure S1.** (**A**) DimPlot – GSE129363 object UMAP-clustering; (**B**) SingleR automatic cell type annotation (Blueprint DB); (**C**) FeaturePlot – UMAP-plot showing principal distribution of *CDH13* gene expression (encoding for T-cadherin) in the GSE129363 object; (**D**) FeaturePlot – UMAP-plot showing principal distribution of *DPP4* gene expression in the GSE129363 object.

Next, we used the 14267219 dataset from the GEO database (NCBI) and analyzed the TS_Skin.h5ad sample (<https://figshare.com/articles/dataset/Tabula_Sapiens_release_1_0/14267219>). This sample corresponded to subcutaneous adipose tissue. Cell type annotation of all clusters was performed using the automatic cell type identification tool (Blueprint DB) (Figure S2). We detected co-localization of cells expressing *DPP4* and *CDH13* in Cluster 0, which was annotated as fibroblasts and adipocytes based on the automatic identification. Additionally, we observed expression of *CDH13* and *DPP4* in endothelial cells within Cluster 1, consistent with previously published data (doi: 10.3389/fendo.2019.00080; doi: 10.1007/s004180100252).


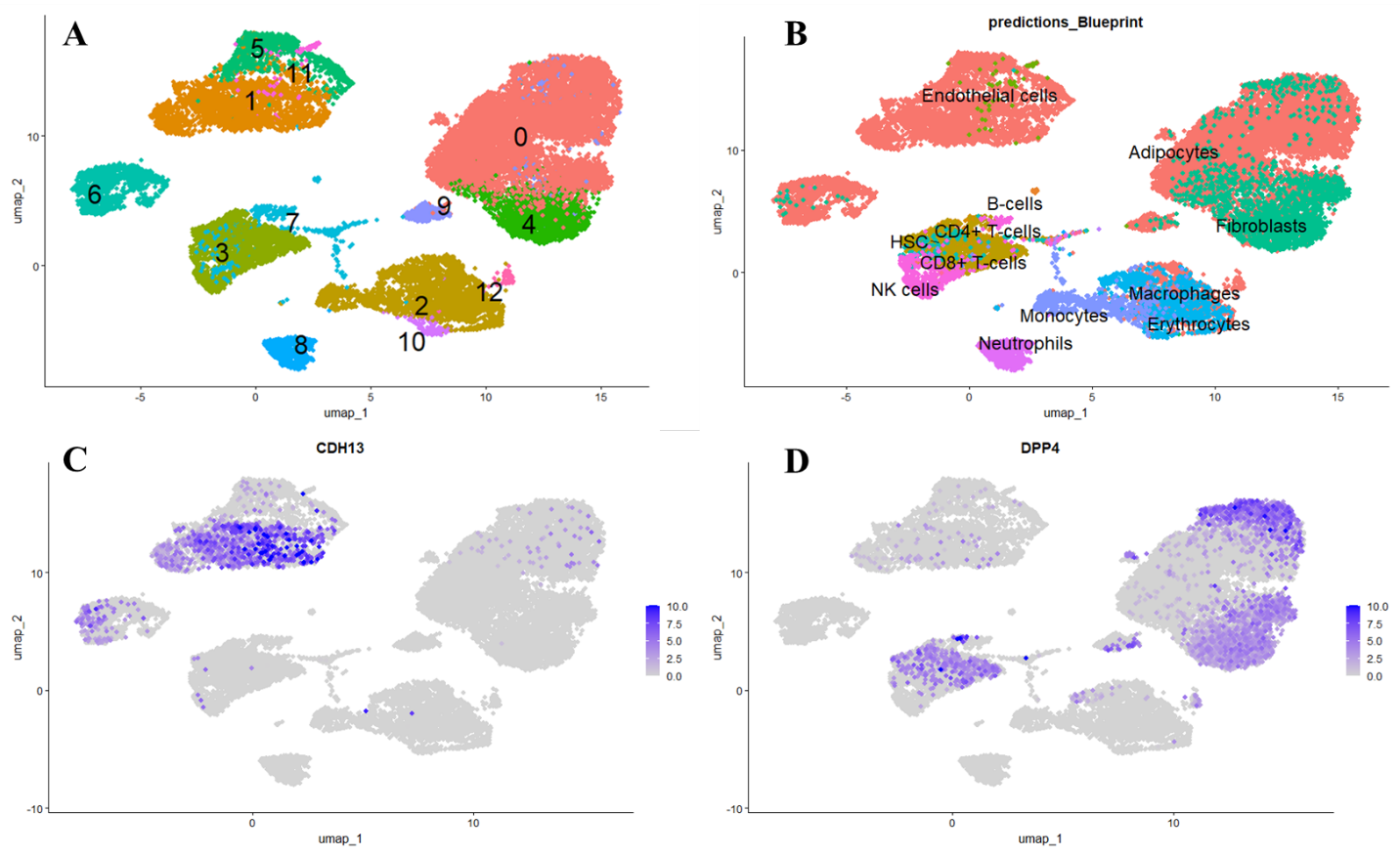


**Figure S2.** (**A**) DimPlot – 14267219 object UMAP-clustering; (**B**) SingleR automatic cell type annotation (Blueprint DB); (**C**) FeaturePlot – UMAP-plot showing principal distribution of *CDH13* gene expression (encoding for T-cadherin) in the 14267219 object; (**D**) FeaturePlot – UMAP-plot showing principal distribution of *DPP4* gene expression (encoding for T-cadherin) in the 14267219 object.

Finally, we analyzed sample GSM5519464 from the GSE182158 dataset, available in the GEO database (NCBI) (https://tabula-sapiens-portal.ds.czbiohub.org/whereisthedatadoi: 10.1002/ctm2.650. PMID: 34965030; PMCID: PMC8715893). We performed the dataset analysis using the R package Seurat v5.0.2. Filtering was conducted using the parameters nFeature_RNA > 1500 & nFeature_RNA < 5000 & nCount_RNA > 5000 & nCount_RNA < 35000 & percent.mt < 5. The first 30 components were analyzed. The FindClusters function was executed to obtain clusters with the parameter resolution = 0.3. Cell type annotation was performed by manually assigning a cell type to each cluster. The FindAllMarkers function for marker identification was run with parameters only.pos = FALSE, min.pct = 0.05, logfc.threshold = 0.05.

The specimen comprised 3 samples obtained from adipose tissue of healthy females who underwent surgical liposuction for cosmetic purposes (Figure S3, S4). We detected co-localization of cells expressing *DPP4* and *CDH13* within Cluster 2, corresponding to fibroblasts according to the automatic identification (Figure S3), which was annotated as fibroblasts using the automatic cell type identification tool (Blueprint DB). Manual cell type annotation further revealed that cells in Cluster 2 also expressed stem-like cell markers (*CD36, NOTCH3, NESTIN, SOX4*), mesenchymal stem cell markers (*PDGFRB, PDGFA, CD44*), and preadipocyte markers (*CEBPG, FABP4, IGFBP5*) according to (Figure S4).


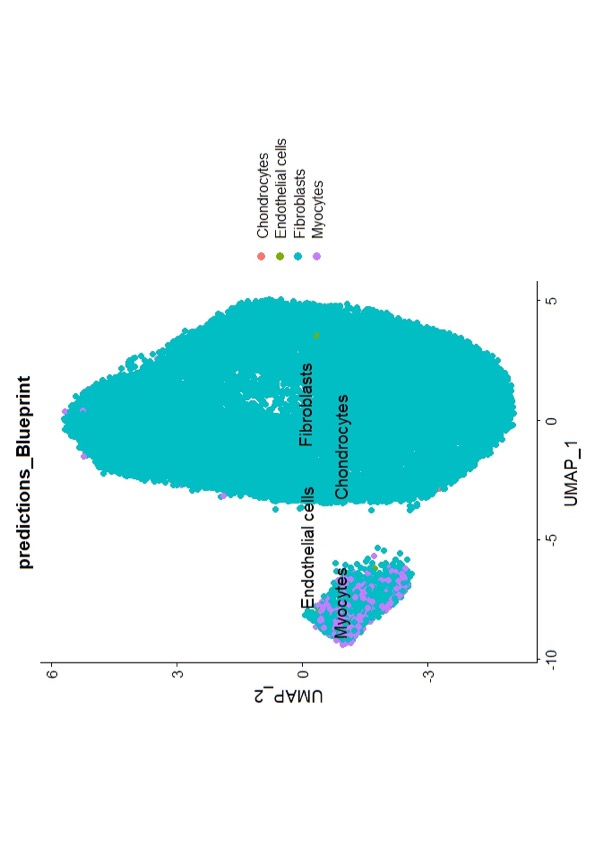


**Figure S3.** SingleR automatic cell type annotation (Blueprint DB).


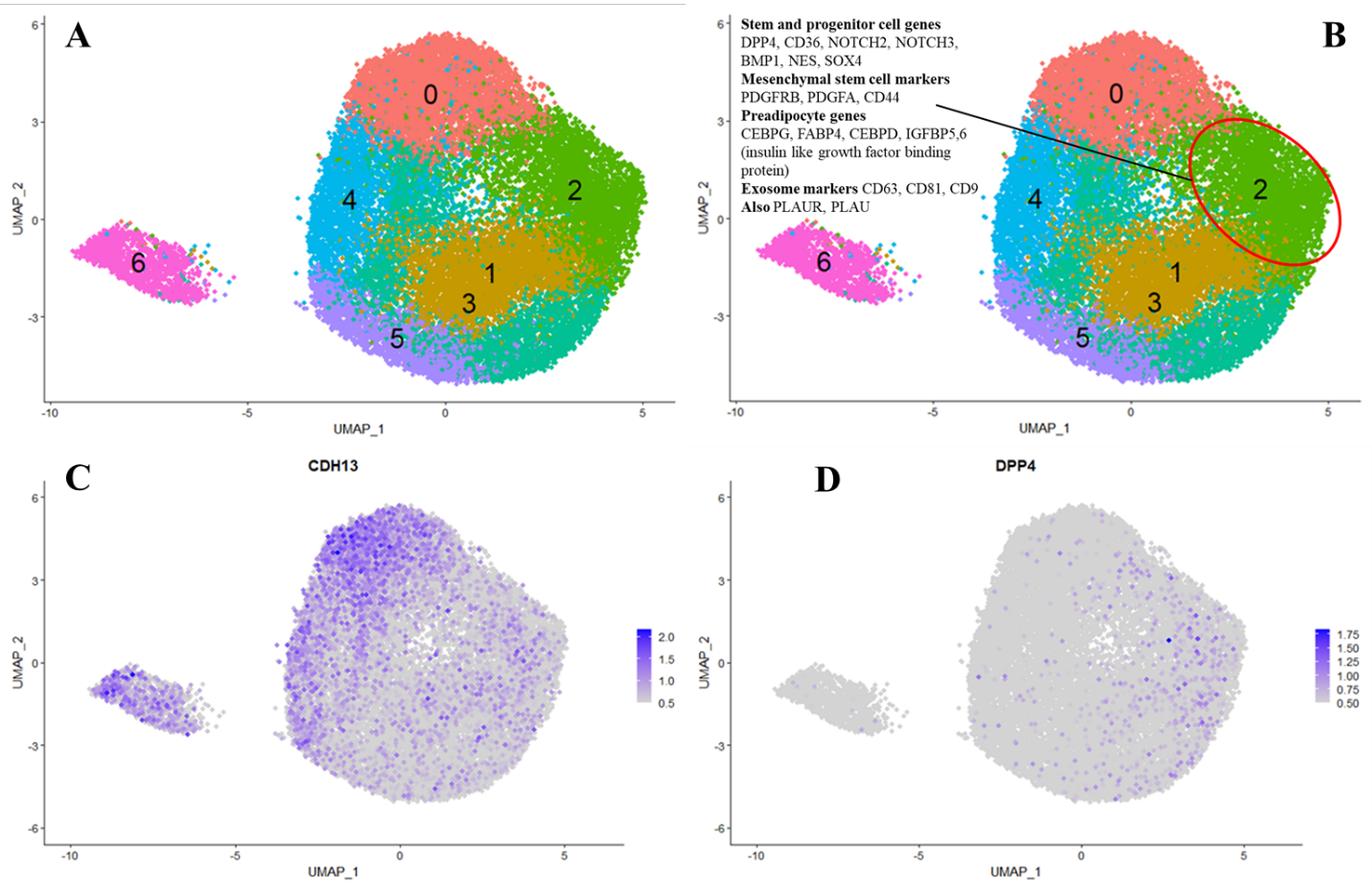


**Figure S4.** (**A**) DimPlot – GSE182158 object UMAP-clustering; (**B**) 2 cluster manual cell type annotation; (**C**) FeaturePlot – UMAP-plot showing principal distribution of *CDH13* gene expression (encoding for T-cadherin) in the GSE182158 object; (**D**) FeaturePlot – UMAP-plot showing principal distribution of *DPP4* gene expression in the GSE182158 object.

Hence, supplementary analysis involving publicly available datasets reinforces our hypothesis that within mesenchymal stem cells (MSCs), specific subpopulations may exhibit stem-like characteristics, encompassing cells that express both *CDH13* and *DPP4*.
